# p53 inhibitor iASPP is an unexpected suppressor of KRAS and inflammation-driven pancreatic cancer

**DOI:** 10.1038/s41418-023-01168-3

**Published:** 2023-06-03

**Authors:** Paul Miller, Elliot H. Akama-Garren, Richard P. Owen, Constantinos Demetriou, Thomas M. Carroll, Elizabeth Slee, Khatoun Al Moussawi, Michael Ellis, Robert Goldin, Eric O’Neill, Xin Lu

**Affiliations:** 1 Ludwig Institute for Cancer Research, Nuffield Department of Clinical Medicine, University of Oxford, Oxford, OX3 7DQ UK; 2 Department of Oncology, University of Oxford, Oxford, OX3 7DQ UK; 3 Centre for Pathology, Department of Medicine, Imperial College London, London, W2 1NY UK

**Keywords:** Cell biology, Tumour-suppressor proteins, Cancer models, Cancer microenvironment

## Abstract

Oncogenic KRAS activation, inflammation and p53 mutation are key drivers of pancreatic cancer (PC) development. Here we report iASPP, an inhibitor of p53, as a paradoxical suppressor of inflammation and oncogenic KRAS^G12D^-driven PC tumorigenesis. iASPP suppresses PC onset driven by KRAS^G12D^ alone or KRAS^G12D^ in combination with mutant p53^R172H^. iASPP deletion limits acinar-to-ductal metaplasia (ADM) in vitro but accelerates inflammation and KRAS^G12D^-induced ADM, pancreatitis and PC tumorigenesis in vivo. KRAS^G12D^/iASPP^Δ8/Δ8^ tumours are well-differentiated classical PCs and their derivative cell lines form subcutaneous tumours in syngeneic and nude mice. Transcriptomically, either iASPP deletion or p53 mutation in the KRAS^G12D^ background altered the expression of an extensively overlapping gene set, comprised primarily of NF-κB and AP1-regulated inflammatory genes. All these identify iASPP as a suppressor of inflammation and a p53-independent oncosuppressor of PC tumorigenesis.

## Introduction

Pancreatic cancer (PC) is a lethal malignancy with a dismal 11% 5-year survival due to advanced disease at presentation and limited treatment options [1]. Genetic studies have identified oncogenic KRAS mutations in approximately 95% of cases, while loss of the tumour suppressor p53 through mutation or deletion occurs in over 50% of cases [2]. Although *TP53* is the most frequently mutated tumour suppressor in PC, it is poorly understood how wild-type (WT) p53 function is bypassed in non-mutated *TP53* PC. As KRAS mutation precedes *TP53* mutation/deletion and cancer onset, it is hypothesised that impairment of p53 activity is required for the development of oncogenic KRAS-driven tumours [3]. A similar hypothesis may also apply to other oncogenic RAS-driven tumours such as colon and lung cancers.

The most studied cellular factors that can inhibit the tumour suppressive function of p53 in WT p53-containing tumours are MDM2 and iASPP (inhibitor of Apoptosis Stimulating Protein of p53). MDM2 is an E3 ubiquitin ligase of p53 that inhibits p53 by both binding to the N-terminal region of p53 and also promoting its proteasome-mediated degradation [4]. iASPP, a member of the ASPP (Ankyrin repeat domain, SH3 domain and Proline rich sequence containing Protein) family of proteins [5], binds the DNA binding domain of p53 and regulates its target selective transcription. RNA-seq and p53 ChIP-seq analyses have confirmed that many iASPP/p53 coregulated targets are involved in regulating apoptosis, consistent with iASPP as an evolutionarily conserved inhibitor of p53-mediated apoptosis [6, 7]. Evidence to date suggests that iASPP is an oncoprotein, as high expression of iASPP associates with poor prognosis in ovarian, melanoma, prostate, glioma and bladder cancers [8–12]. In vitro and xenograft studies have shown that iASPP has pro-proliferative and chemoresistant properties. These observations led to the hypothesis that iASPP deficiency would enhance WT p53 activity to inhibit tumorigenesis [13].

Pancreatitis, especially hereditary pancreatitis, is a major risk factor for PC [14]. In response to pancreatitis-related cellular stress and inflammatory signalling, exocrine acinar cells undergo acinar-to-ductal metaplasia (ADM), which is thought to be an initiating event in pancreatic tumorigenesis [15]. ADM requires the activation of the TGFα/EGFR/RAS/MAPK pathway: TGFα overexpressing mice exhibit spontaneous ADM, while TGFα stimulation of acinar explants causes ADM in the absence of oncogenic KRAS [16, 17]. Pancreas-specific (Ptf1a-Cre) EGFR and ADAM17 knockout mice effectively eliminate oncogenic RAS-induced ADM and tumorigenesis [18]. Interestingly, iASPP-deficient mice share overlapping developmental phenotypes with EGFR, TGFα and ADAM17 mutant mice, suggesting that iASPP may play a role similar to that of EGFR or TGFα in inflammation and oncogenic RAS-induced ADM [19–22]. All these support the hypothesis that iASPP could be an oncogene that counteracts the tumour suppressive function of WT p53 in PC tumorigenesis. To test this, we examined the ability of iASPP to regulate the pancreatic inflammatory response and PC formation in oncogenic KRAS-driven pancreatitis and PC models using pancreas-specific (Pdx1-Cre) compound transgenic mice expressing mutant KRAS in the presence or absence of iASPP (exon-8 deletion). The ability of iASPP to influence PC development was also investigated in both WT and mutant p53 backgrounds.

## Results

### iASPP unexpectedly suppresses oncogenic KRAS-driven PC onset

**Fig. 1:**
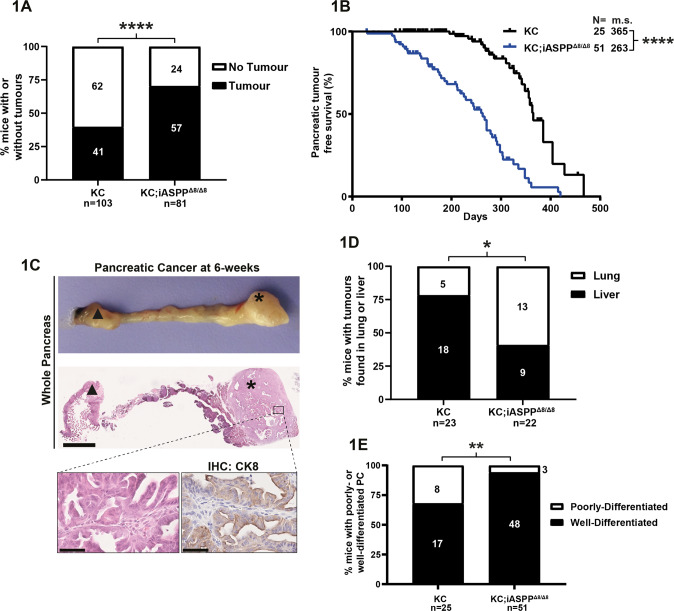
iASPP unexpectedly suppresses oncogenic KRAS-driven PC onset. **A** Percentage of mice with evidence of tumour (pancreatic, visceral, or subcutaneous; excluding lymphoma) from the KC (*n* = 103) and KC;iASPP^Δ8/Δ8^ (*n* = 81) overall survival cohort. **** = *p* < 0.0001; n.s. = not significant; Fisher’s exact test. **B** Kaplan-Meier pancreatic tumour free survival of KC and KC;iASPP^Δ8/Δ8^ mice in the overall survival cohort. Number of mice and median survival for tumour-bearing mice with non-pancreatic tumour bearing mice censored. **** = *p* < 0.0001, log-rank test. *N* = , number of mice; m.s., median survival. **C** Pancreatic cancer at 6 weeks in KC;iASPP^Δ8/Δ8^ mouse. Upper image, dissected pancreas with attached duodenum. Middle image, whole pancreas HE stained. Bottom left image, well-differentiated HE stained PC. Bottom right image, IHC stained with cytokeratin 8 to demonstrate epithelial original. Middle image scale bar, 1 mm. Bottom images scale bar, 50 µm. Duodenum is denoted by black triangle and PC is denoted asterisk. **D** Percentage of extra-pancreatic tumours that are either lung or liver in the KC and KC;iASPP^Δ8/Δ8^ survival cohort. * = *p* < 0.05; n.s., not significant; Fisher’s exact test. **E** Percentage of primary pancreatic tumours in which the cancer is either predominantly well- or poorly-differentiated in the KC and KC;iASPP^Δ8/Δ8^ survival cohort. ** = *p* < 0.01; Fisher’s exact test.

We investigated the effect of iASPP on tumour development in the presence of WT p53 using Pdx1-Cre to express oncogenic KRAS^G12D^ (KC) alone or concurrently delete iASPP to generate KC and KC;iASPP^Δ8/Δ8^ mice respectively (Supplementary Fig. S1A). According to the animal license requirement, all mice in the survival cohort that reached a pre-defined endpoint were culled. Therefore, all-cause mortality analysis includes mortality caused by pancreatic and extra-pancreatic pathology such as upper gastrointestinal tract hyperplasia, benign facial and genital papillomas, and thymic lymphomas as reported previously [23, 24] All-cause mortality analysis revealed that median overall survival (OS) for KC;iASPP^Δ8/Δ8^ (*n* = 81) and KC mice (*n* = 103) was 179 versus 260 days (Supplementary Fig. S1B), a significant reduction in KC;iASPP^Δ8/Δ8^ cohort. For all mice, the pancreas and other organs with suspected macroscopic disease were assessed histologically for the presence of tumours (Supplementary Fig. S1C). Within the time frames examined and accounting for all tumours detected, a significantly higher percentage of tumour incidences were observed in KC;iASPP^Δ8/Δ8^ than in KC mice (Fig. 1A). Histological evidence of ≥1 pancreatic, visceral, or subcutaneous tumours showed that tumour free survival (TFS) for KC;iASPP^Δ8/Δ8^ and KC mice were 244 and 356 days respectively (Supplementary Fig. S1D). Thoracic lymphomas are often considered as an off-target effect of oncogenic KRAS and were excluded from subsequent survival and tumour-spectrum analyses [25]. Lymphomas were detected in both KC and KC;iASPP^Δ8/Δ8^ cohorts and accounted for 27% of histologically diagnosed KC tumours (Supplementary Fig. S1E, F). By excluding lymphomas it becomes clear that pancreatic tumour incidence is similar between KC and KC;iASPP^Δ8/Δ8^ cohorts (Supplementary Fig. S1G). However, even under this analysis condition, median pancreatic TFS for KC;iASPP^Δ8/Δ8^ and KC cohorts was 263 versus 365 days respectively with the earliest PC found at 6 weeks (Fig. 1B, C), a significant reduction in pancreatic TFS in KC;iASPP^Δ8/Δ8^ cohort. All these showed that iASPP-deletion accelerated oncogenic KRAS^G12D^-driven PC onset.

Most mice developed tumours at a single site but some developed additional extra-pancreatic tumours (Supplementary Fig. S1H). Extra-pancreatic tumours, most likely metastatic PC, were predominantly found in the liver or lungs, but the KC cohort also exhibited subcutaneous tumours or splenic disease (Supplementary Fig. S1I). Extra-pancreatic tumours in the lung versus liver was ~60%:40% in KC;iASPP^Δ8/Δ8^ mice and ~25%:75% in KC mice (Fig. 1D). In mice with a histologically confirmed PC, there is an increase in extra-pancreatic tumour incidence, presumed metastases, in KC mice compared to KC;iASPP^Δ8/Δ8^ (Supplementary Fig. S1J). Notably, KC;iASPP^Δ8/Δ8^ mice had a greater proportion of well-differentiated PC compared to the KC mice, and the infrequent spindle cell morphology was only found in the KC cohort (Fig. 1E and Supplementary Fig. S1K). All these support iASPP as an important suppressor of PC initiation but not progression. Furthermore, iASPP is a suppressor of well-differentiated PC with a metastatic tropism that favours lung over liver.

### iASPP suppresses oncogenic KRAS-driven PC independently of p53

As KC mice contain WT p53, we tested iASPP’s ability to suppress PC tumorigenesis independently of p53 in KPC mice which have concurrent heterozygous mutations of the KRAS and p53 that accelerate tumour onset and metastasis [26]. We crossed the KC and KC;iASPP^Δ8/Δ8^ mice with mice containing a p53^R172H^ allele (equivalent to human p53 hotspot mutation R175H) to generate KPC (Pdx1-Cre; Kras^G12D/+^; p53^R172H/+^) (*n* = 48) and KPC;iASPP^Δ8/Δ8^ (*n* = 15) cohorts (Supplementary Fig. S2A). All-cause mortality analysis showed that the median OS was significantly lower in the KPC;iASPP^Δ8/Δ8^ than in the KPC cohorts (102 versus 148 days) (Fig. S2B). Mice with thymic lymphomas were excluded for subsequent survival analyses (KPC (*n* = 1), KPC;iASPP^Δ8/Δ8^ (*n* = 0)) (Fig. S2C). Tumour-free survival showed that KPC;iASPP^Δ8/Δ8^ mice had a significantly lower median survival (103 days) than KPC mice (155 days) (Fig. S2D).

**Fig. 2:**
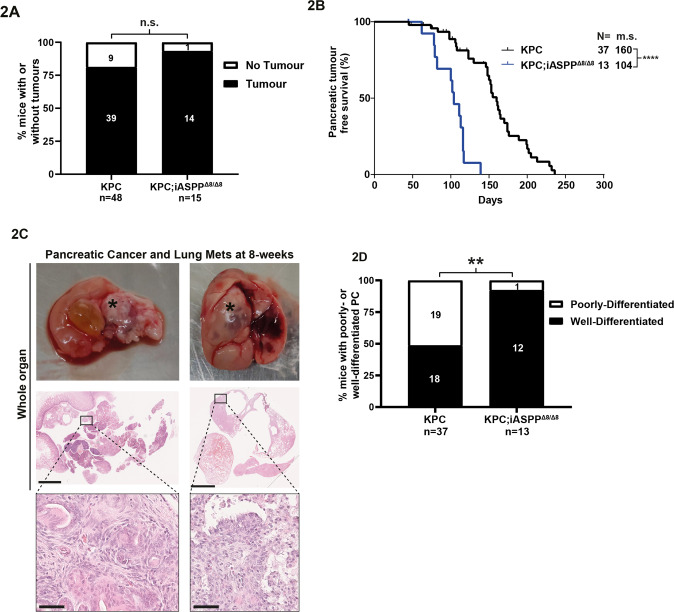
iASPP suppresses oncogenic KRAS-driven PC independently of p53. **A** Percentage of mice with evidence of tumour (pancreatic, visceral, or subcutaneous, excluding lymphoma) from the KPC (*n* = 48) and KPC;iASPP^Δ8/Δ8^ (*n* = 15) overall survival cohort. n.s. = not significant; Fisher’s exact test. **B** Kaplan-Meier pancreatic tumour free survival of the KPC and KPC;iASPP^Δ8/Δ8^ mice in the overall survival cohort. **** = *p* < 0.0001; log-rank test. *N* = , number of mice; m.s., median survival. **C** Metastatic PC at 8 weeks in KPC;iASPP^Δ8/Δ8^ mouse. Upper images; left, dissected pancreas with attached duodenum; right, dissected lung with cystic tumour. Asterisks denote tumours. Middle images; left, whole pancreas HE stained; right, whole lung HE stained. Lower images; left, focus of PC; right, PC metastases in the lung. Middle image left scale bar, 2.5 mm. Middle image right scale bar, 5 mm. Bottom images scale bar, 50 µm. **D** Percentage of PC in which the predominant lesion is well- or poorly-differentiated in the KPC (*n* = 37) and KPC;iASPP^Δ8/Δ8^ (*n* = 13) pancreatic TFS cohort. ** = *p* < 0.01; Fisher’s exact test.

Comparing KPC and KPC;iASPP^Δ8/Δ8^ cohorts, we observed small differences in the percentage of mice with evidence of tumours at necropsy (81% versus 93%, Fig. 2A) or the proportion of pancreatic tumours (97% versus 93%, Supplementary Fig. S2E). However, pancreatic TFS was reduced in KPC;iASPP^Δ8/Δ8^ mice (104 days) compared to KPC mice (160 days) (Fig. 2B). Primary pancreatic disease was the most common diagnosis at necropsy in both KPC and KPC;iASPP^Δ8/Δ8^ cohorts with similar proportions of mice with extra-pancreatic tumours, presumed metastatic disease (Supplementary Figure S2F). Only single sites of extra-pancreatic disease were found in KPC;iASPP^Δ8/Δ8^ mice, whereas KPC mice had up to two sites of disease in addition to pancreatic primary tumours (Supplementary Fig. S2G). Extra-pancreatic tumours were present in the liver, lung, spleen, diaphragm, and subcutaneously in KPC mice (Supplementary Fig. S2H). Notably, the KPC;iASPP^Δ8/Δ8^ cohort only displayed extra-pancreatic tumours in the lung and subcutaneously. Early metastatic disease was present and lung deposits were identifiable with concurrent primary tumours from as early as 8-weeks in the KPC;iASPP^Δ8/Δ8^ cohort (Fig. 2C).

Histological examination of tumour types identified well- and poorly-differentiated PC, with occasional examples of anaplastic cancer in both KPC and KPC;iASPP^Δ8/Δ8^ (Supplementary Fig. S2I). However, most KPC;iASPP^Δ8/Δ8^ cancers were predominantly well-differentiated, whereas KPC was almost equally split between well- and poorly differentiated and only KPC had examples of spindle cell morphology (Fig. 2D). Taken together, accelerated tumour onset and the development of predominantly well-differentiated cancers in KPC;iASPP^Δ8/Δ8^ mice are consistent with iASPP being a potent suppressor of PC initiation but not progression, even in the KPC background. It also demonstrated that iASPP can suppress PC development independently of p53.

### Inflammation induces iASPP expression in vivo and iASPP mRNA level associates with good prognosis in human classical PC

Inflammatory conditions such as hereditary pancreatitis predisposes to PC development [27] and one of the most well-known mouse pancreatitis models is induced by intraperitoneal injections of the cholecystokinin analogue caerulein [28]. Previous studies showed that p53 expression is induced in pancreatitis [29] and by caerulein injection [30]. As an inhibitor of p53 and a recently identified suppressor of inflammation [31, 32], we tested whether caerulein treatment can also induce iASPP expression. Pancreatic lysates were derived from WT mice at 0, 1 and 2 days post-caerulein treatment as indicated (Supplementary Fig. S3A, top panel). Similar to p53, we observed upregulation of iASPP in caerulein-treated pancreatic lysates compared to PBS-treated control lysates, demonstrating that inflammatory stimuli induce iASPP expression in vivo (Supplementary Fig. S3A, bottom panel).

**Fig. 3:**
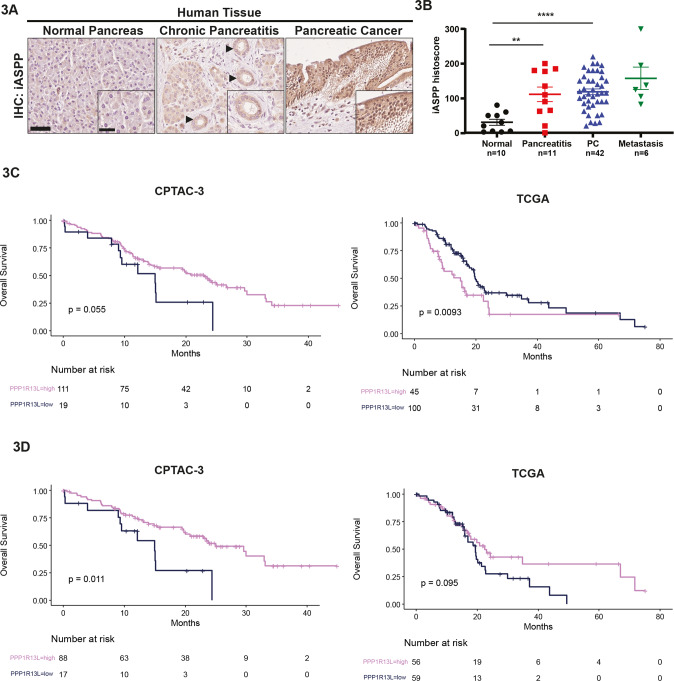
Inflammation induces iASPP expression in vivo and high iASPP mRNA associates with good prognosis of classical PC. **A** Human pancreas iASPP staining. iASPP expression in normal pancreas (left), chronic pancreatitis (centre), and pancreatic cancer (right). Ductal metaplasia denoted by black arrows. Scale bar, 100 µm (inset scale bar, 40 µm). **B** iASPP histoscore in relation to human tissue histology; normal (*n* = 10), pancreatitis (*n* = 11), pancreatic cancer (PC) (*n* = 42) and metastasis (*n* = 6) from pancreatic primary tissue. Error bars represent mean ± SEM, and data were analysed by unpaired t-test. ** = *p* < 0.01, **** = *p* < 0.0001. **C** Kaplan-Meier analysis for overall survival plot in CPTAC-3 and TCGA cohorts with high and low iASPP (PPP1R13L) mRNA expression cut-offs defined using maxstat R package. **D** Kaplan-Meier analysis for overall survival plot in classical (consensus) PC samples in the CPTAC-3 and TCGA cohorts with high and low iASPP (PPP1R13L) mRNA expression cut-offs defined using maxstat R package.

We examined iASPP expression levels between normal and diseased pancreas using immunohistochemical (IHC) staining of human patient samples obtained from surgical sections and tissue arrays. We observed increased iASPP expression in chronic pancreatitis samples (including early ductal lesions) and pancreatic cancer, relative to normal pancreas (Fig. 3A, B). In surgical tumour sections, increased iASPP is detected in PC and in the tumour-adjacent acini cells relative to those acini cells which are distant from the tumour (Supplementary Fig. S3B). A similar expression pattern was seen for p53 (Supplementary Fig. S3B). Notably, we also observed similar iASPP and p53 expression patterns in KC mice with PC: increased iASPP and p53 were detected in the tumour-adjacent areas relative to acini distant from the PC (Supplementary Fig. S3C). These results are consistent with iASPP as a suppressor of inflammation.

Increased expression of iASPP has been associated with poor prognosis in multiple cancers, including prostate, melanoma, and glioma [10–12]. We thus investigated the association of iASPP mRNA expression and clinical outcomes in two publicly available human PC cohorts (CPTAC-3 [33] and TCGA [34]). No association between OS and iASPP mRNA expression was identified in the CPTAC-3 cohort, and high iASPP levels were associated with poor prognosis in the TCGA cohort, as expected (Fig. 3C). Consensus transcriptomic analyses of human PC cohorts identify two subtypes: classical and squamous, associated with well- and poorly-differentiated PC, respectively [35]. Knowing iASPP-deletion associates with histologically well-differentiated cancers, we re-analysed OS in human PC cases expressing a classical-associated transcriptomic phenotype. Interestingly, high iASPP mRNA expression associates with good prognosis in the CPTAC-3 cohort with classical subtypes. Similarly, there is a trend between high iASPP mRNA and better prognosis in the TCGA dataset (Fig. 3D). The notion that inflammation induces iASPP expression in vivo and high iASPP mRNA level associated with good prognosis of classical PC are in agreement with iASPP being a suppressor of classical PC development.

### iASPP is a paradoxical suppressor of oncogenic KRAS- and inflammation-induced ADM in vivo

**Fig. 4:**
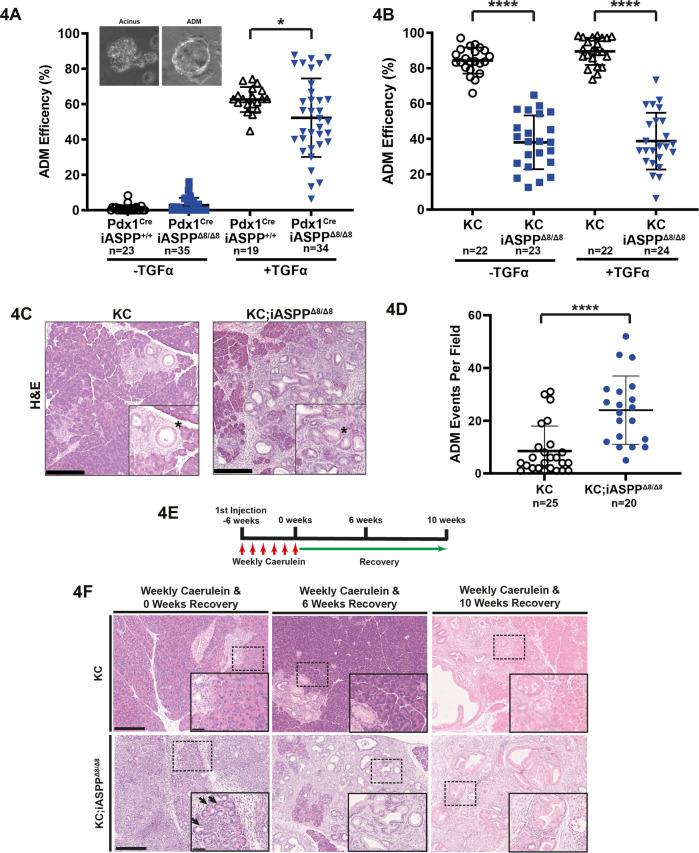
iASPP is a paradoxical suppressor of oncogenic KRAS- and inflammation-induced ADM in vivo. **A** Acinar-to-ductal metaplasia efficiency on day 5 of cultured Pdx1-Cre; iASPP^+/+^ (*n* = 2) and Pdx1-Cre;iASPP^Δ8/Δ8^ (*n* = 3) pancreatic acini with and without TGFα stimulation. * = *p* < 0.05, using unpaired t-test. Inset images in graph are phase contrast of ADM assay. Acinus (left) with no evidence of ductal metaplasia and acinus having undergone ADM with ring morphology (right). **B** Acinar-to-ductal metaplasia efficiency on day 5 of cultured KC (*n* = 2) and KC;iASPP^Δ8/Δ8^ acini (*n* = 3) pancreatic acini with and without TGFα stimulation. **** = *p* < 0.0001 using unpaired t-test. **C** H&E staining of representative 12-week old KC and KC;iASPP^Δ8/Δ8^ normal acini and ADM (denoted by asterisk). Scale bar, 250 µm. **D** Quantification of ADM events per field in young (12- to 16-week old) KC (*n* = 5) and KC;iASPP^Δ8/Δ8^ (*n* = 4) mice. **** = *p* < 0.0001 using Mann-Whitney test. **E** Injection schedule of weekly caerulein (250 µg/kg) over 6 weeks followed by planned culling after 0-, 6- and 10-week recovery. **F** Representative H&E images of KC and KC;iASPP^Δ8/Δ8^ pancreas following weekly caerulein after 0-, 6-, and 10-weeks recovery. Inset image is magnified dotted box. Black arrows indicate ADM in progress. Scale bars, 250 µm (main images) and 50 µm (inset images).

ADM is induced by direct stimulation of the TGFα-EGFR-MAPK signalling pathway [16, 18], and is the first biological process initiating PC development. We thus tested iASPP’s ability to suppress ADM both ex vivo and in vivo. Acinar explants from 6-week-old Pdx1-Cre;iASPP^+/+^ and Pdx1-Cre;iASPP^Δ8/Δ8^ mice (Supplementary Fig. S4A) were cultured for 5 days in the presence or absence of TGFα, a ligand that can bind EGFR to stimulate RAS/MAPK signalling, and the frequency of ADM was assessed by the proportion of acini that converted to ductal morphology after 5 days (Fig. 4A, inset). The dot plot shows that in the absence of TGFα, very few ADM events were detected. With TGFα stimulation, ~60–70% acini from Pdx1-Cre;iASPP^+/+^ mice exhibited ADM, whereas there is a large variation in the ability of Pdx1-Cre;iASPP^Δ8/Δ8^ acini to undergo ADM, with medium ADM frequency to be significantly lower than that observed with Pdx1-Cre;iASPP^+/+^ acini (Fig. 4A). Acinar explants from 6-week old KC and KC;iASPP^Δ8/Δ8^ mice were similarly cultured for five days in the presence or absence of TGFα. KC explants showed higher ADM frequency than that in KC;iASPP^Δ8/Δ8^ explants (~80% versus ~40%) regardless of additional TGFα stimulation (Fig. 4B). These showed that iASPP is required for efficient ADM ex vivo in the context of oncogenic KRAS and TGFα stimulation.

Reprogramming of acinar cells into ductal-like cells can be a transient response to pancreatic injury and a persistent response to oncogenic KRAS [15]. We thus compared ADM events in KC;iASPP^Δ8/Δ8^ and KC mice. Unexpectedly, in 12-week-old mouse pancreata without histological evidence of PC, KC;iASPP^Δ8/Δ8^ mice had a higher number of spontaneous ADM events compared to KC mice (Fig. 4C, D).

To investigate whether iASPP suppresses inflammation-induced ADM in vivo, we induced moderate acute pancreatitis in mice through 8 hourly intraperitoneal injections (50 µg/kg per injection) of caerulein on consecutive two days (Supplementary Fig. S4B). Acute pancreatitis can be temporally divided into three stages post caerulein injection: acute inflammation (from 3 to 36 h), regeneration (from day 2 to 6) and refinement (from day 7 to 14) [36]. We first induced acute pancreatitis in germline iASPP^Δ8/Δ8^ [37], iASPP^Δ8/+^, and iASPP^+/+^ mice, referred to as iASPP-KO, -HET and -WT mice respectively (Supplementary Fig. S4C). On day 12, iASPP-WT mice exhibited morphologically normal acini with no evidence of oedema or inflammatory infiltrates. Pancreata in heterozygous iASPP-HET mice were morphologically like iASPP-WT but exhibited small foci of ADM with inflammatory infiltrates. However, iASPP-KO mice exhibit a severe phenotype with reduced pancreatic mass, loss of most normal acini, and an abundance of ductal-like structures consistent with ADM.

Tissue response to pancreatic inflammation relies on the interplay between acini, fibroblasts, and immune cells; thus, it is difficult to deduce the cellular drivers of ADM in germline iASPP-KO mice. We tested iASPP function in acinar cells, the purported PC cell-of-origin, and assessed this using Pdx1-Cre;iASPP^Δ8/Δ8^ mice in the acute pancreatitis and recovery model. No histological differences were observed between the control PBS or caerulein-injected pancreata after 2-, 7-, and 14 days recovery (Supplementary Fig. S4D, E). This suggests that the tissue response to pancreatitis may be controlled by iASPP functioning in multiple pancreatic cell types.

Given the incongruous ADM phenotype between germline and Pdx1-Cre-specific iASPP-knockout mice, we investigated the contribution of iASPP to ADM, driven by the synergy of oncogenic KRAS and inflammation. We induced a mild form of chronic pancreatitis by single weekly intraperitoneal caerulein injections for six weeks in KC and KC;iASPP^Δ8/Δ8^ mice, assessing the tissue response at 0-, 6- and 10-weeks of recovery (Fig. 4E, F). Interestingly, while KC pancreata were histologically similar at each time point to non-injected mice (data not shown), the KC;iASPP^Δ8/Δ8^ pancreata exhibited profound ADM that persisted after 6- and 10 weeks of recovery. In the absence of oncogenic KRAS mutation, Pdx1-Cre;iASPP^+/+^ and Pdx1-Cre;iASPP^Δ8/Δ8^ pancreata exhibited no ADM immediately after weekly caerulein injection or after 18 weeks of recovery (Supplementary Fig. S4F, G). These data suggest that iASPP is a paradoxical suppressor of ADM as it is required for ADM ex vivo but it inhibits inflammation and oncogenic RAS-driven ADM in vivo. This suggests that iASPP may function via non-cell-autonomous signalling to suppress acinar reprogramming in vivo.

### iASPP regulates its surrounding immune microenvironment in response to pancreatitis and oncogenic KRAS

**Fig. 5:**
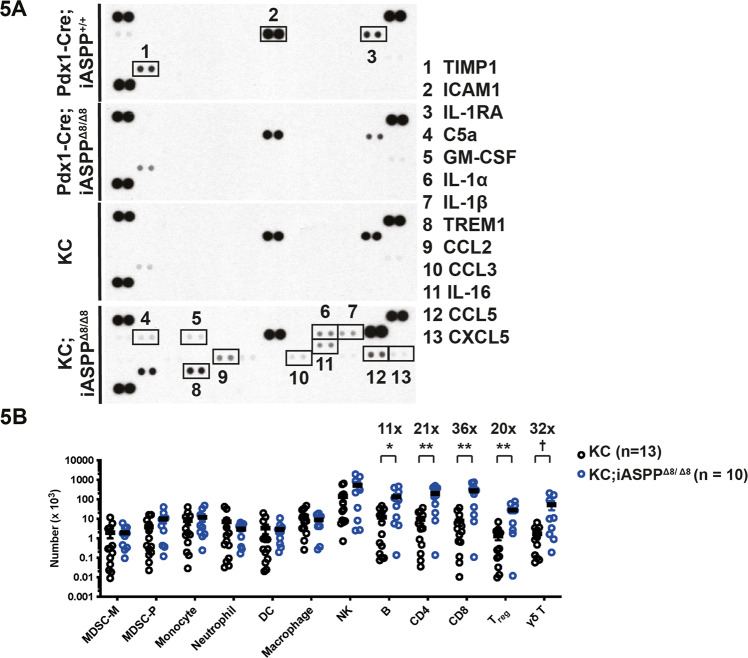
iASPP regulates its surrounding immune microenvironment in response to inflammation and oncogenic KRAS. **A** Cytokine protein ELISA array was performed on murine pancreatic tissue lysates following six weekly doses of caerulein. **B** Immune cell profiling of KC (*n* = 13) and KC;iASPP^Δ8/Δ8^ (*n* = 10) pancreatic tissue samples following six weekly doses of caerulein determined by flow cytometry. Mean number of immune cells per pancreas ± SEM is shown by horizontal lines. MDSC-M: CD11b + Ly6C + Ly6Glo, MDSC-P: CD11b + Ly6cloLy6G+, Monocyte: CD11b + Ly6GloLy6Clo, Neutrophil: CD11b + Ly6G + Ly6C+, DC: CD19-CD11c + MHCII + , Macrophage: CD19-CD68 + F4/80+, NK: CD19-CD49b+, B: CD19+, CD4: CD45 + CD3 + CD4+, CD8: CD45 + CD3 + CD8+, Treg: CD45 + CD3 + CD4 + FoxP3+, γδT: CD45 + CD3 + TCRγδ+. ** *p* = 0.015, * *p* < 0.01, † *p* = 0.042. 11x, 21x, 36x, 20x and 32x signifies fold change in KC;iASPP^Δ8/Δ8^ relative to KC. Statistical analysis was carried out by multiple unpaired *t* tests.

Given the paradoxical in vivo and ex vivo phenotypes described above in early pancreatic neoplastic transformation, we next examined the inflammatory response to iASPP deletion. We used a cytokine array to investigate the presence of immunomodulatory factors in tissue lysates of Pdx1-Cre;iASPP^+/+^, Pdx1-Cre;iASPP^Δ8/Δ8^, KC and KC;iASPP^Δ8/Δ8^ pancreata after 6 weeks of mild chronic pancreatitis (Fig. 5A). Minor differences were apparent between Pdx1-Cre;iASPP^+/+^, Pdx1-Cre;iASPP^Δ8/Δ8^ and KC. However, KC;iASPP^Δ8/Δ8^ pancreatic lysates had higher levels of cytokines that are known to modulate pancreatic tumorigenesis including GM-CSF, IL-1α, IL-1β, CCL2, CCL5 and other inflammatory mediators such as TIMP-1 and TREM-1. A pro- or anti-tumorigenic tissue microenvironment and the milieu of inflammatory mediators are controlled by proportions of immune cells and stromal cells with further variations in cell-specific activation and signalling. To characterise the immune cell profile of the iASPP-deficient oncogenic KRAS tissues following pancreatitis, fluorescence-activated cell sorting was performed on whole pancreatic lysates. There was increased infiltration of B, CD4+, CD8+, Treg and γδT cells in KC;iASPP^Δ8/Δ8^ pancreata following chronic pancreatitis relative to KC (Fig. 5B). Differences in the immune cell profile were not present in the spleen suggesting that the immune cell phenotype was dependent on pancreatic iASPP status (Supplementary Fig. S5A). These results are consistent with the observation that iASPP may regulate ADM via a non-cell-autonomous pathway.

### iASPP suppresses PC via cell intrinsic and extrinsic pathways

To understand how iASPP may suppress PC development in vivo and to determine whether iASPP-deficient tumour growth is regulated by a host immune response, we generated cell lines from various PC tumours derived from KC (*n* = 3) and KC;iASPP^Δ8/Δ8^ (*n* = 3) mice. Sanger sequencing confirmed that all KC and KC;iASPP^Δ8/Δ8^ cell lines expressed WT p53 and iASPP deletion was confirmed by western blot (Supplementary Fig. S6A, B). All KC and KC;iASPP^Δ8/Δ8^ cell lines were treated with Nutlin to stabilise p53 and to induce p53-targets p21 and Bax. A control KPC cell line confirmed mutant p53 is not increased with Nutlin and does not induce p21 or Bax expression (Supplementary Fig. S6C).

**Fig. 6:**
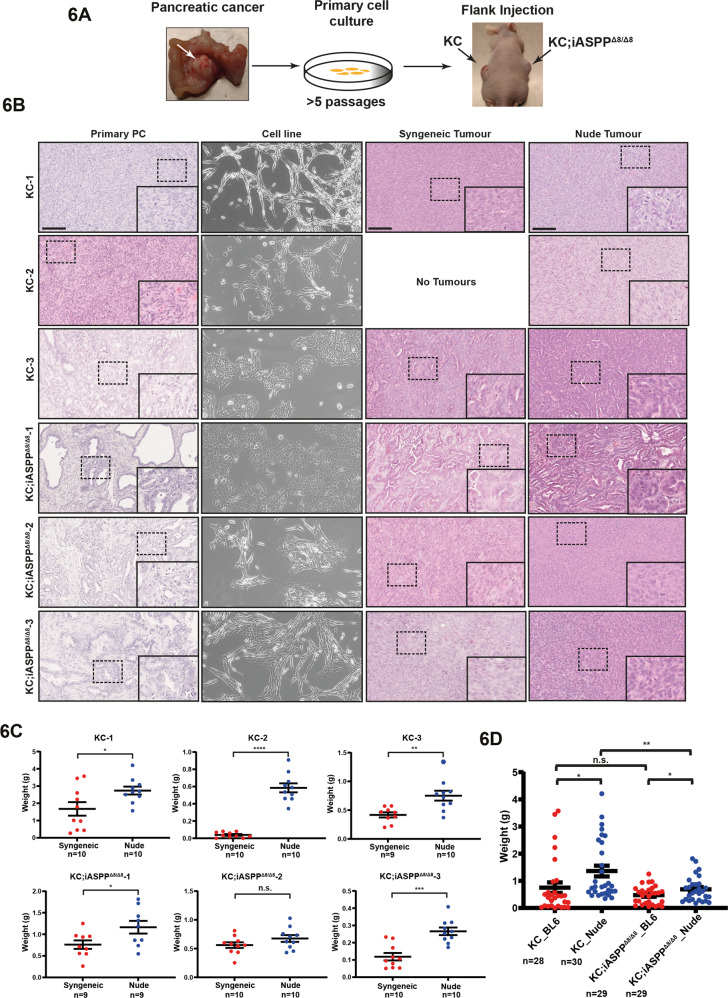
iASPP suppresses PC growth through both cell intrinsic and extrinsic pathways. **A** Schematic diagram for the experiment; dissection of PC (left), derivation of primary PC cell lines (centre) and tumours at culling following bilateral flank subcutaneous injections (right). **B** Left column, representative images of primary PC from three KC and three KC;iASPP^Δ8/Δ8^ mice used to generate cell lines. Second left column, representative phase contrast imaging of corresponding primary pancreatic cancer cell lines. Second right column, representative images of excised syngeneic-injected tumour. Right column, representative images of excised nude-injected tumour. Scale bars 100 µm. **C** Mean weights ± SEM of syngeneic and nude subcutaneous tumours for KC-1, KC-2, KC-3 and KC;iASPP^Δ8/Δ8^-1, KC;iASPP^Δ8/Δ8^-2, KC;iASPP^Δ8/Δ8^-3 cell lines. **** = *p* < 0.0001; *** = *p* < 0.001; ** = *p* < 0.01; * = *p* < 0.05; n.s., not significant. *P* values were calculated using unpaired t-test. **D** Average weights for KC and KC;iASPP^Δ8/Δ8^ cell lines tumours in BL6 and nude mice. ** = *p* < 0.01; * = *p* < 0.05; n.s., not significant. *P* values were calculated using unpaired t-test.

The PC-derived cell lines were subcutaneously injected into the flanks of immunocompetent (syngeneic C57BL/6) and immunocompromised (nude) mice. For each mouse, one flank was injected with KC cells while the contralateral flank was injected with KC;iASPP^Δ8/Δ8^ cells (Fig. 6A). Primary cancer cell cultures KC-1 and KC-2 were derived from poorly differentiated pancreatic cancers and were morphologically more mesenchymal with spindle-shaped cells. KC-3 was derived from a moderately- to well-differentiated primary tumour with an epithelial cobblestone-like morphology. Notably, subcutaneous (SC) tumours from KC-1 and KC-2 cell lines retained a poorly differentiated morphology, whereas those from KC-3 reconstituted ductal features (Fig. 6B, syngeneic and nude tumour panels). KC;iASPP^Δ8/Δ8^-1, -2 and -3 primary cultures were derived from moderately- to well-differentiated primary tumours, with KC;iASPP^Δ8/Δ8^-1 having a typical epithelial appearance in culture and KC;iASPP^Δ8/Δ8^-2 and -3 having a mixed appearance. SC tumours from KC;iASPP^Δ8/Δ8^-1 retained ductal morphology, while KC;iASPP^Δ8/Δ8^-2 and KC;iASPP^Δ8/Δ8^-3 had more poorly differentiated features than that of KC;iASPP^Δ8/Δ8^-1 and the primary tumours they originated from. These results suggest that the morphology of flank-injected tumours aligns with the in vitro morphology of the respective primary cell cultures and the in vivo spontaneous tumours from which they were derived from.

Tumour weight varied significantly between each cell line with KC-2 failing to initiate a tumour in syngeneic mice and KC-1 producing the largest (average weight > 1.5 g) (Fig. 6C). All SC tumours had higher average weights in nude mice than in C57BL6 mice except KC;iASPP^Δ8/Δ8^-2, in agreement with the fact that immunocompetent mice were able to elicit an anti-tumour adaptive immune response. On average, there was no significant difference in tumour weight between KC and KC;iASPP^Δ8/Δ8^ induced SC tumours in C57BL6 mice, but KC tumours grew better in nude mice than KC;iASPP^Δ8/Δ8^ tumours (Fig. 6D). These data suggest that iASPP is likely to suppress PC development via a combination of cell-intrinsic and extrinsic pathways in a tissue context-dependent manner. In vivo and in a correct tissue environment, such as the pancreas, the tumour suppressive function of iASPP is more pronounced.

### iASPP deletion or p53 mutation on a KC background induces profound transcriptional overlap on inflammatory genes

**Fig. 7:**
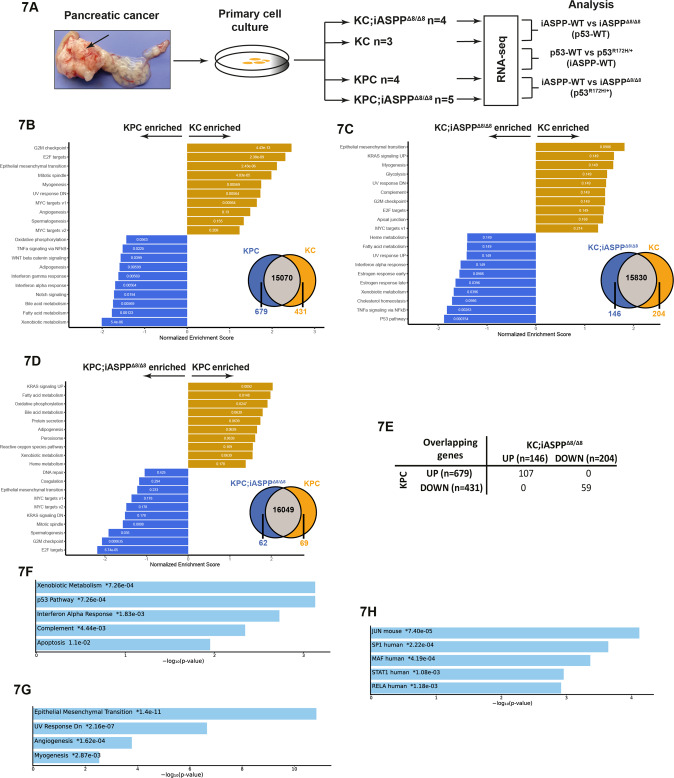
iASPP deletion or p53 mutation on a KC background induces profound transcriptional overlap of inflammatory genes. **A** Schematic diagram for the derivation of primary pancreatic cancer cell lines and RNA-seq analysis for KC, KC;iASPP^Δ8/Δ8^, KPC, and KPC;iASPP^Δ8/Δ8^ mice. **B** GSEA for KPC (blue) and KC (orange) and number of enriched genes in KPC and KC cell lines. **C** GSEA for KC;iASPP^Δ8/Δ8^ (blue) and KC (orange) and number of enriched genes in KC;iASPP^Δ8/Δ8^ and KC cell lines. **D** GSEA for KPC;iASPP^Δ8/Δ8^ (blue) and KPC (orange) and number of enriched genes in KPC;iASPP^Δ8/Δ8^ and KPC cell lines. **E** Overlapping upregulated and downregulated DEGs in KC;iASPP^Δ8/Δ8^ and KPC cell lines relative to KC. **F** Top 5 hallmark gene sets enriched for overlapping upregulated genes in the KC;iASPP^Δ8/Δ8^ and KPC DEGs. **G** Top 4 hallmark gene sets enriched for overlapping downregulated genes in the KC;iASPP^Δ8/Δ8^ and KPC DEGs. **H** Top 5 transcription factors represented by overlapping genes from upregulated KC;iASPP^Δ8/Δ8^ and KPC DEGs from the TRRUST transcription factor database.

To gain insight into how iASPP may suppress oncogenic RAS-driven PC, we performed RNA sequencing (RNA-seq) of PC cell lines with the following genotypes: KC (*n* = 3), KC;iASPP^Δ8/Δ8^ (*n* = 4), KPC (*n* = 4) and KPC;iASPP^Δ8/Δ8^ (*n* = 5) (Fig. 7A). All cell lines used in Fig. 6 were included. Analysis of transcriptomes of KPC and KC cell lines identified 1140 differentially expressed genes (DEGs) (679 upregulated and 431 downregulated) in the KPC/p53^R172H^ versus KC/p53-WT expressing cells (Fig. 7B). Gene set enrichment analysis (GSEA) showed increased expression of metabolic pathways (xenobiotic, fatty acid, and bile acid metabolism) in KPC cells (Fig. 7B and Supplementary Fig. S7A, B). In contrast, KC cells showed increased expression of cell-cycle (G2M checkpoint, E2F targets and mitotic spindle) and epithelial–mesenchymal transition (EMT) genes. Metabolic pathways are associated with the classical subtype of PC, and enrichment of EMT and E2F targets are features of basal-like PC, consistent with KPC and KC cell lines sharing features of classical and basal-like PCs, respectively [38].

Comparing transcriptomes of KC;iASPP^Δ8/Δ8^ versus KC cells revealed 350 DEGs (146 upregulated and 204 downregulated) in KC;iASPP^Δ8/Δ8^. Consistent with previous studies [6, 31], KC;iASPP^Δ8/Δ8^ transcriptomes showed enrichment of DEGs in p53 and TNFα inflammatory signalling pathways (Fig. 7C and Supplementary Fig. S7C, D) whereas KC transcriptomes were again enriched in EMT and cell-cycle related genes. Moreover, metabolic pathways were upregulated in KC;iASPP^Δ8/Δ8^ compared to KC cells in agreement with iASPP being a suppressor of classical PC. Only 131 DEGs (62 upregulated and 69 downregulated) were found between KPC and KPC;iASPP^Δ8/Δ8^, in agreement with iASPP being a potent inhibitor of p53 transcription. Upregulated genes observed in KPC;iASPP^Δ8/Δ8^ cells compared to KPC cells are cell cycle-related genes (E2F targets, G2M checkpoint, MYC targets) (Fig. 7D and Supplementary Fig. S7E, F).

Surprisingly, when comparing overlapping DEGs in KPC (mutant p53) and KC;iASPP^Δ8/Δ8^ (WT p53), we identified no genes that were up in KC;iASPP^Δ8/Δ8^ and down in KPC or vice versa (Fig. 7E). In contrast, we identified 107 DEGs that are upregulated in both KC;iASPP^Δ8/Δ8^ and KPC cells in comparison to KC cells. Specifically, 73% of upregulated DEGs (107/146) were also found in KPC cells. Similarly, 29% downregulated KC;iASPP^Δ8/Δ8^ DEGs (59/204) are common with KPC cells. The extensive overlap of up and downregulated genes in KPC and KC;iASPP^Δ8/Δ8^ relative to KC cells suggest that iASPP deletion and p53 mutation on the KC background may acquire a common mechanism to bypass WT p53’s tumour suppressive function.

GSEA of overlapping DEGs between KC;iASPP^Δ8/Δ8^ and KPC cells revealed that genes involved in p53/apoptosis, interferon response, and xenobiotic metabolism were significantly enriched (Fig. 7F). Conversely, among the commonly downregulated DEGs, the EMT pathway was the most enriched (Fig. 7G), consistent with KPC and KC;iASPP^Δ8/Δ8^ cells having features of classical PCs. Finally, analysis of the 96 commonly upregulated DEGs identified AP1 (JUN) and NF-κB (RELA) among the top 5 TFs that can regulate the expression of the identified DEGs (Fig. 7H).

Knowing that iASPP is an inhibitor of inflammation and a binding partner and inhibitor of p65RelA/NF-κB [39] and JunB/JunD/AP1 [31], we tested whether iASPP may control the expression levels and cellular localisations of p65RelA or JunB/JunD to inhibit the NF-κB or AP1 induced inflammatory response in vivo. We performed immunohistochemical staining of AP1 components JunB and JunD and phosphorylated p65RelA (p-p65RelA, a transcriptionally active form of p65RelA) in mouse PC sections (Supplementary Fig. S7G–I). In KC, KC;iASPP^Δ8/Δ8^, and KPC tissues, JunB, JunD and nuclear p-p65RelA are not expressed in morphologically normal acini but are readily detectable in metaplastic lesions and pancreatic cancer cells. However, iASPP status had minimal impact on the levels and cellular localisations of JunB, JunD and p-p65RelA in the PC sections examined. Although the precise mechanisms by which iASPP suppresses NF-κB and AP1 transcription remains unknown, the data shown in this study illustrate that iASPP is a potent suppressor of inflammation and that, unexpectedly, iASPP deletion and p53 mutation in KC background use overlapping NF-κB and AP1 transcriptional pathways to induce inflammation.

## Discussion

The tumour suppressor p53 is mutated in around 50% of human cancers. A key question is how p53 loses its tumour suppressive function in tumours containing wild-type p53. In an attempt to address this challenge, we unexpectedly found that iASPP, a known p53 inhibitor, is a paradoxical suppressor of inflammation and PC initiation. iASPP suppresses PC onset driven by either oncogene KRAS or by a combination of oncogenic KRAS^G12D^ and p53^R172H^, demonstrating its ability to suppress PC initiation independently of p53.

PC can be classified into classical-PC or squamous/basal-like, hereafter termed basal-like, subtypes based on transcriptomic features that inform response to chemotherapy and prognosis [35]. Classical PCs express many pancreatic developmental genes (*PDX1*, *KLF5*, *FOXA3* etc.) and keratins (19, 8, 18), whereas basal-like PCs express ΔNp63 targets and EMT genes that are associated with enhanced metastatic potential. Basal-like PCs are more metastatic compared to classical PCs which often exhibit well-differentiated phenotypes [40]. Interestingly, transcriptomic analysis of cell lines derived from PCs with various genotypes revealed that iASPP deletion or p53 mutation on the KRAS^G12D^ background induced profound expression of overlapping gene sets, many of which are found in classical PCs. This provides molecular evidence for how iASPP deletion induces well-differentiated classical PCs relative to KC mice, in agreement with our in vivo findings in both spontaneous and subcutaneous tumours. All these established iASPP as a suppressor of classical PCs and it can perform its suppressor function in a cell-intrinsic manner.

Although reduced survival in mouse PC models is often associated with poorly differentiated and highly metastatic cancers, high mortalities can also be caused by accelerated onset of well-differentiated PCs. For example, KC mice expressing mutant p53 (R172H/+; KPC) or deleted p53 (KC;p53null) have a similar median survival, but KPC mice develop markedly increased metastatic disease at necropsy compared to that of KC;p53null mice [41]. Similar to KC;p53-null, KC;iASPP^Δ8/Δ8^ mice have significantly reduced OS but exhibit no enhancement of metastatic potential compared to KC mice. Spontaneous PCs developed in KC;iASPP^Δ8/Δ8^ mice have higher propensity to develop lung metastasis than liver metastasis. This unusual metastatic organotropism is distinct from that observed in KPC or KC mice. Although it remains unclear how iASPP deletion can cause such a strong bias towards lung metastasis, it is consistent with iASPP as a tumour suppressor of well-differentiated PCs. Patients with lung metastasis PC tend to have a better prognosis than ones with liver metastasis [42, 43]. Intriguingly, classical PC expressing high iASPP is associated with good prognosis.

Elevated TGFα/EGFR/RAS/MAPK signalling and inflammation induce ADM in the pancreas to promote the formation of PanIN and PC development. Hence, suppressors of inflammation and/or key players of TGFα/EGFR/RAS/MAPK signalling pathways are likely to inhibit the formation of ADM, PanIN and PC initiation. In addition to binding and inhibiting p53, iASPP was also identified as a binding partner and inhibitor of p65RelA-NF-κB [39]. Recent identification of iASPP as a binding partner of JunB/D-AP1 places iASPP as a downstream effector of TGFα/EGFR/RAS/MAPK signalling pathway [31]. The findings that iASPP is required for TGFα/EGFR/RAS/MAPK-induced ADM in vitro but inhibits KRAS^G12D^-driven ADM in vivo suggest that the paradoxical suppressor role of iASPP is unlikely to be solely mediated through TGFα/EGFR/RAS/MAPK signalling pathway.

The existing data do not allow us to conclude whether iASPP favours the AP1 over NF-κB pathway, vice versa or via a combination of pathways. p65RelA is a key component of NF-κB signalling and is a known oncogene in haematological malignancies. Like many oncogenes and tumour suppressors, the oncogenic and tumour suppressive roles of p65RelA are highly tissue context-dependent [44]. Importantly, in an oncogenic KRAS-driven PC mouse model, similar to our present study, deletion of p65RelA accelerated the formation of ADM, PanINs and PC [45], a set of phenotypes similar to those observed following iASPP deletion in this study. However, concurrent deletion of p65RelA and p53 or p16, to abrogate oncogene-induced senescence, resulted in longer survival [45]. All these suggest that in the oncogenic KRAS-driven mouse PC model iASPP, as an inhibitor of p65RelA, is unlikely to modulate tumour initiation and progression solely via p65RelA.

**Fig. 8:**
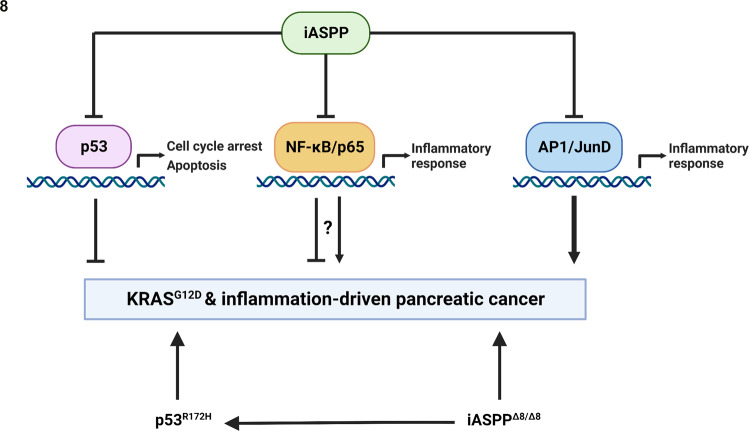
Schematic representation of the tumour suppressive role of iASPP in PC. iASPP is a paradoxical suppressor of tumour initiation in PDAC: although iASPP binds to and inhibits previously-identified tumour suppressive p53 and NF-κB/p65 pathways, iASPP additionally binds to JunD/AP1 and inhibits this inflammatory, oncogenic axis. Whilst the NF-κB/p65 pathway has been shown to be tumour suppressive, it is possible that its upregulation of the inflammatory response confers a dual and conflicting role in contributing to tumour initiation. Mutation of p53 (p53^R172H^) or deletion of iASPP (iASPP^Δ8/Δ8^) on a KRAS^G12D^ background leads to cancer initiation and likely does so through a similar pathway, as evidenced by the altered expression of an extensively overlapping gene set, comprised primarily of NF-κB and AP1-regulated inflammatory genes. The absence of iASPP in addition to p53 mutation leads to an acceleration of KRAS^G12D^-driven PDAC onset.

Recent studies showed that iASPP deletion has a more profound effect on the AP1 pathway than the NF-κB pathway [31]. While we failed to identify any study that reports a specific deletion of JunD in an oncogenic KRAS-driven mouse PC model, a recent transgenic mice study reported that JunD is required in oncogenic KRAS-driven mouse lung tumour model [46]. AP1 is a key mediator of pancreatic inflammatory signalling and sensitises the pancreas to inflammatory stress and oncogenic KRAS signals [47, 48]. A recent study [32] showed that iASPP deletion accelerates oncogenic KRAS-driven lung tumorigenesis. Taken together, iASPP-JunB/D-AP1 axis might have a more profound impact on suppressing PC development than that of the iASPP-p65RelA-NF-κB axis (Fig. 8). Nonetheless, there are extensive functional cross-talks between NF-κB and AP1 and future studies are needed to pinpoint the precise signalling pathways that mediate iASPP’s tumour suppressive function.

The fact that all cell lines derived from KC and KC;iASPP^Δ8/Δ8^ PC tumours contain both WT p53 sequence and transcriptional activity (Fig. S6C) supports the notion that iASPP deletion can accelerate PC onset without the need to mutate p53. Observed upregulation of the p53 signalling pathway in KC;iASPP^Δ8/Δ8^ cells compared to that in KC cells further support the WT p53 status and inhibitory function of iASPP in these cells. Surprisingly, KPC and KC;iASPP^Δ8/Δ8^ cell lines showed profoundly overlapping transcriptomes. Many of the altered gene sets are inflammatory-related gene targets of NF-κB and AP1. Despite unknown molecular mechanisms, both mutant p53 and iASPP can inhibit p53 transcription [6, 49] and previous studies have established extensive crosstalk between WT p53 and NF-κB and AP1 pathways. Thus, iASPP and mutant p53 may act similarly to selectively inhibit the expression of the same set of WT p53-NF-κB or/and p53-AP1 co-regulated target genes. Alternatively, the observed changes may be mediated via a p53-independent but directly NF-κB or/and AP1-dependent pathway. iASPP can bind and inhibit both NF-κB and AP1 through its ability to directly bind p65RelA and JunB/D. The same DNA binding defective mutant p53, p53^R175H^(human)/p53^R172H^(mouse), can activate NF-κB and accelerate inflammation-associated colon cancer [50, 51]. As iASPP can bind both WT and mutant p53 [6], it is possible that mutant p53 may bind iASPP, preventing iASPP from inhibiting NF-κB and AP1 activities. This hypothesis places iASPP downstream of mutant p53 and is consistent with the observation that iASPP deletion can accelerate PC onset in KPC mice.

Regardless, the present study identifies iASPP as a paradoxical suppressor of inflammation and oncogenic KRAS-driven PC tumorigenesis. The transcriptomic overlap of enhanced inflammatory genes caused by either iASPP deletion or p53 mutation supports the idea that elevated inflammation is a common mechanism for bypassing p53 tumour suppression. The results also present a tantalising possibility that mutant p53 might need to inhibit iASPP function to promote inflammation and tumorigenesis.

## Materials and methods

### Resources table

**Table:** 

REAGENT or RESOURCE	SOURCE	IDENTIFIER
Antibodies		
p53	Leica Biosystems	Cat# NCL-L-p53-CM5p
iASPP	Ascites	LXO49.3
β-Tubulin	Santa Cruz	Cat# sc-5274
p21	Santa Cruz	Cat# sc-471
Actin	Santa Cruz	Cat# sc-47778
Bax	Cell Signaling Technology	Cat# 2772 S
Cytokeratin 8	DHSB	Cat# TROMA-I
Biotinylated Anti-Mouse antibody	Vector Labs	Cat# BA-9200-1.5
Biotinylated Anti-Rabbit antibody	Vector Labs	Cat# BA-1000-1.5
Biotinylated Anti-Rat antibody	Vector Labs	Cat# BA-9400-1.5
APC-Cy7 anti-CD3 (17A2)	BioLegend	Cat# 100221
PE-Cy5 anti-CD4 (RM4-5)	BioLegend	Cat# 100513
BV421 anti-CD4 (GK1.5)	BioLegend	Cat# 100437
FITC anti-CD4 (RM4-5)	eBioscience	Cat# 11-0042-82
APC anti-CD8α (53-6.7)	BioLegend	Cat# 100711
BV510 anti-CD8α (53-6.7)	BioLegend	Cat# 100751
BV785 anti-CD8α (53-6.7)	BioLegend	Cat# 100749
BV510 anti-CD11b (M1/70)	BioLegend	Cat# 101245
PerCP-Cy5.5 anti-CD11b (M1/70)	BioLegend	Cat# 101227
BV421 anti-CD11c (N418)	BioLegend	Cat# 117329
PE-Cy7 anti-CD11c (N418)	BioLegend	Cat# 117317
BV650 anti-CD19 (6D5)	BioLegend	Cat# 115541
BV510 anti-CD25 (PC61)	BioLegend	Cat# 102041
FITC anti-CD45 (30-F11)	BioLegend	Cat# 103107
PE/Dazzle 594 anti-CD49b (DX5)	BioLegend	Cat# 108923
PE-Cy7 anti-CD68 (FA-11)	BioLegend	Cat# 137015
BV421 anti-F4/80 (BM8)	BioLegend	Cat# 123131
BV711 anti-F4/80 (BM8)	BioLegend	Cat# 123147
BV785 anti-Ly6C (HK1.4)	BioLegend	Cat# 128041
PerCP-Cy5.5 anti-Ly6G (1A8)	BioLegend	Cat# 127615
Alexa Fluor 700 anti-MHCII (M5/114.15.2)	BioLegend	Cat# 107621
PE anti-TCRγδ (UC7-13D5)	BioLegend	Cat# 107507
Biological Samples		
Tissue Microarray of Human Pancreatic Disease	US Biomax	Cat# PA2081b
Chemicals, peptides, and recombinant proteins		
Rat Tail Collagen I	Gibco	Cat# A1048301
HBSS (10X)	Gibco	Cat# 14065056
RPMI 1640 Medium	Gibco	Cat# 11875093
Collagenase P	Roche	Cat# 11213857001
Soybean Trypsin Inhibitor (STI)	Sigma-Aldrich	Cat# T6522
Dexamethasone	Sigma-Aldrich	Cat# D4902
TGFα	PeproTech	Cat# 100-16 A
Caerulein	Sigma-Aldrich	Cat# C9026
Matrigel Basement Membrane Matrix	Corning	Cat# CLS356234
Nutlin-3a	Sigma-Aldrich	Cat# SML0580
Permeabilization Buffer	eBioscience	Cat# 00-8333-56
Critical Commercial Assays		
Mouse Cytokine Array Panel A - Proteome Profiler	R&D systems	Cat# ARY006
RNeasy Mini Kit	QIAGEN	Cat# 74106
RNase-Free DNase Set	QIAGEN	Cat# 79254
QuantSeq FWD 3’ mRNA-Seq Library Prep Kit	Lexogen	Cat# 015
Deposited Data		
Mouse pancreatic cell line RNA-seq	This paper	Raw data: deposited with GEO with accession GSE226595
Experimental Models: Cell lines		
KC-1: Pdx1-Cre; Kras^G12D/+^; p53^R172H/+^; Sex Female	This paper	N/A
KC-2: Pdx1-Cre; Kras^G12D/+^; p53^R172H/+^; Sex Male	This paper	N/A
KC-3: Pdx1-Cre; Kras^G12D/+^; p53^R172H/+^; Sex Female	This paper	N/A
KC;iASPP^Δ8/Δ8^-1: Pdx1-Cre; Kras^G12D/+^; iASPP^Δ8/Δ8^; Sex Female	This paper	N/A
KC;iASPP^Δ8/Δ8^-2: Pdx1-Cre; Kras^G12D/+^; iASPP^Δ8/Δ8^; Sex Male	This paper	N/A
KC;iASPP^Δ8/Δ8^-3: Pdx1-Cre; Kras^G12D/+^; iASPP^Δ8/Δ8^; Sex Male	This paper	N/A
KC;iASPP^Δ8/Δ8^-4: Pdx1-Cre; Kras^G12D/+^; iASPP^Δ8/Δ8^; Sex Male	This paper	N/A
KPC-1: Pdx1-Cre; Kras^G12D/+^; p53^R172H/+^; Sex Male	This paper	N/A
KPC-2: Pdx1-Cre; Kras^G12D/+^; p53^R172H/+^; Sex Female	This paper	N/A
KPC-3: Pdx1-Cre; Kras^G12D/+^; p53^R172H/+^; Sex Female	This paper	N/A
KPC-4: Pdx1-Cre; Kras^G12D/+^; p53^R172H/+^; Sex Male	This paper	N/A
KPC;iASPP^Δ8/Δ8^-1: Pdx1-Cre; Kras^G12D/+^; p53^R172H/+^; iASPP^Δ8/Δ8^; Sex Male	This paper	N/A
KPC;iASPP^Δ8/Δ8^-2: Pdx1-Cre; Kras^G12D/+^; p53^R172H/+^; iASPP^Δ8/Δ8^; Sex Female	This paper	N/A
KPC;iASPP^Δ8/Δ8^-3: Pdx1-Cre; Kras^G12D/+^; p53^R172H/+^; iASPP^Δ8/Δ8^; Sex Male	This paper	N/A
KPC;iASPP^Δ8/Δ8^-4: Pdx1-Cre; Kras^G12D/+^; p53^R172H/+^; iASPP^Δ8/Δ8^; Sex Male	This paper	N/A
KPC;iASPP^Δ8/Δ8^-5: Pdx1-Cre; Kras^G12D/+^; p53^R172H/+^; iASPP^Δ8/Δ8^; Sex Male	This paper	N/A
Experimental Models: Organisms/Strains		
Mouse: C57BL/6J females (6-8 weeks)	Envigo	Cat# 57
Mouse: Athymic Nude-*Foxn1*^*nu*^ (6-8 weeks)	Envigo	Cat# 69
Mouse: KC; *Pdx1-Cre*; *Kras*^LSL-G12D/+^	[23]	N/A
Mouse: KPC; *Pdx1-Cre*; *Kras*^LSL-G12D/+^; *p53*^*R172H/+*^	[26]	N/A
Mouse: *iASPP*^*Δ8/Δ8*^	[37]	N/A
Mouse: KC;iASPP^Δ8/Δ8^; *Pdx1-Cre*; *Kras*^LSL-G12D/+^; *iASPP*^*Δ8/Δ8*^	This paper	N/A
Mouse: KPC;iASPP^Δ8Δ8^; P*dx1-Cre*; *KRAS*^+/LSL-G12D^; p53^R172H/+^; iASPP^Δ8/Δ8^	This paper	N/A
Software and Algorithms		
GraphPad Prism 8.3	GraphPad Prism Software	https://www.graphpad.com/scientific-software/prism/
NDP.scan version 2.8.24	Hamamatsu, Japan	https://www.hamamatsu.com/eu/en/product/life-science-and-medical-systems/digital-slide-scanner/U12388-01.html
NDP.view2 version 2.8.24	Hamamatsu, Japan	https://www.hamamatsu.com/eu/en/product/life-science-and-medical-systems/digital-slide-scanner/U12388-01.html
FlowJo	FlowJo	https://www.flowjo.com/solutions/flowjo/downloads
Enrichr	Ma’ayan Lab	https://maayanlab.cloud/Enrichr/
TCGABiolinks 2.24.3	Bioconductor R package	https://academic.oup.com/nar/article/44/8/e71/2465925?login=true
edgeR 3.38.0	Bioconductor R package	https://academic.oup.com/bioinformatics/article/26/1/139/182458?login=true
GSVA 1.44.0	Bioconductor R package	https://bmcbioinformatics.biomedcentral.com/articles/10.1186/1471-2105-14-7
Cluster 2.1.3	CRAN	https://cran.r-project.org/web/packages/cluster/cluster.pdf
Survival 3.3-1	CRAN	https://link.springer.com/book/10.1007/978-1-4757-3294-8
Survminer 0.4.9	CRAN	https://rpkgs.datanovia.com/survminer/index.html
R 4.2.1	Vienna, Austria	https://www.R-project.org/
DESeq2	CRAN	https://genomebiology.biomedcentral.com/articles/10.1186/s13059-014-0550-8
pheatmap	CRAN	https://CRAN.R-project.org/package=pheatmap
EnhancedVolcano	Github	https://github.com/kevinblighe/EnhancedVolcano
ashr	CRAN	10.1093/biostatistics/kxw041

### Animals

All mice were housed and all procedures were performed at the Wellcome Trust Centre for Human Genetics at the University of Oxford. All animal studies described in this study were performed in accordance with guidelines provided by the University of Oxford Institutional Animal Care and Use Committee. All procedures were performed under the Home Office Animal Scientific Procedures Act 1986 guidelines (PPL PP7833295). The number of mice used in the survival cohorts was not pre-specified.

Generation of iASPP transgenic mice in which the loxP-flanked exon 8 of PPP1R13L, is deleted by CMV-Cre, has been previously described [37]. Compound mutant mice were generated by crossing *PPP1R13L*^*loxP/loxP*^ (iASPP^∆8/∆8^) C57BL/6 mice with previously described LSL-KRAS^G12D^ [52], Pdx1-Cre [23], LSL-Trp53^R172H^ [53], and LSL-Rosa26EYFP [54].

After mice were weaned, DNA was isolated from ear clips by incubation in TDB (50 mM KCl, 10 mM Tris-HCl, 0.1% Triton-X100 in H_2_O) with 0.4 mg/mL Proteinase K (Qiagen) overnight at 55 °C, then 10 min at 95 °C. Pdx1-Cre mice were genotyped using the primers: Cre-F, 5′-CATTTGGGCCAGCTAAACAT-3′; Cre-B, 5′-ATTCTCCCACCGTCAGTACG-3′. iASPP^∆8/∆8^ mice were genotyped using the following primers: FRANT9, 5′-GGGTAGGAAAAAGGGCTGAG-3′; FLP2, 5′-CCGAATTGGAGAAGTGAAGC-3′. LSL-KRAS^G12D/+^ mice were genotyped using the following primers: Y116, 5′-TCCGAATTCAGTGACTACAGATG-3′; Y117, 5′CTAGCCACCATGGCTTGAGT-3′; Y118, 5′-ATGTCTTTCCCCAGCACAGT-3′. Trp53LSL-R172H/+ mice were genotyped using the following primers: p53u, 5′-CTTGGAGACATAGCCACACTG-3′, p53wt, 5′-TTACACATCCAGCCTCTGTGG-3′; and p53mut, 5′-AGCTAGCCACCATGGCTTGAGTAA 3′.

### ADM assay

ADM assay was performed as previously described [55]. Briefly, pancreata from Pdx1-Cre;iASPP^+/+^, Pdx1-Cre;iASPP^Δ8/Δ8^, KC and KC;iASPP^Δ8/Δ8^ mice were harvested, washed in ice-cold HBSS, and minced with dissection scissors. Acini were dissociated in 10 mg/ml collagenase P for 20 min at 37 °C with agitation then centrifuged 2000 rpm for 2 min at 4 °C. The pellet was washed three times in cold HBSS and 5% FBS and then passed through a 100 µm filter and pelleted at 1000 rpm for 2 min at 4 °C. The cell pellet was resuspended in 3D-culture medium (RPMI 1640, 1% FBS, 1% Pen/Strep, 0.1 mg/ml STI, 1 µg/ml Dexamethasone) and mixed with equal parts collagen then immediately plated onto collagen-coated wells. 3D-culture media was changed on days 1 and 3. 3D-culture media was supplemented with 50 ng/ml TGFα when required. Acini were analysed on day 5 for quantification of ADM efficiency.

### Tumour challenge

Mouse PC-derived cells were mixed with equal volumes of Matrigel and subcutaneously injected into the right and left flanks of 8–10 weeks old male C57BL/6J and nude mice at 1 × 10^6^ cells/mouse. Mice were not randomised. A group size of at least ten tumours per condition was assumed to achieve significantly different results (*p* = 0.05) with a power of 80%. Tumours were monitored and mice were taken at humane endpoint as dictated by the animal license or on day 21 from injection. Tumours were dissected, weighed, and processed for histological staining. Tumour analysis was not performed blinded.

### Mouse pancreatic cancer cell line generation

In brief, mouse tumours were dissected, washed in PBS, and minced with scissors on 35 mm culture dishes. Floating tissue was aspirated away and maintained in DMEM containing 10% FBS and 100 IU/ml penicillin/streptomycin supplemented with glutamine. Established primary cultures were passaged at least five times to remove fibroblast contamination. Cells were maintained in a humidified incubator at 37^o^C with 5% CO_2_. All cell lines were negative for mycoplasma.

### Pancreatitis induction

Acute pancreatitis (AP) was induced by two sets of 8 hourly intraperitoneal (i.p.) caerulein injections (50 µg/kg) over 2 consecutive days. Chronic pancreatitis (CP) was induced by weekly single i.p. caerulein injections (250 µg/kg) for six consecutive weeks. For both AP and CP, the final day of caerulein injection was considered day 0. Mice were culled at various time points as stated in the results.

### Haematoxylin and Eosin (H&E) staining

Tissues were fixed overnight in 10% formalin, embedded in paraffin, and cut into 4 µm sections. Paraffin sections were deparaffinised, rehydrated in a gradient of ethanol (100%, 90%, 70%, 30%) and washed in water. Slides were incubated in Harris haematoxylin for 3 min. Slides were rinsed in running tap water to remove excess stain and differentiated in 1% acidic alcohol for 10 s. Slides were washed again in tap water and immersed in Scott’s water for 30 s. Slides were rinsed in tap water and stained in eosin for 4 mins. Slides were then washed in water to remove excess eosin and dehydrated with an increasing gradient of ethanol solutions. Slides were cleared using histoclear, and mounted with non-aqueous mounting medium (Vectamount, Vector Labs) and coverslips.

### Nutlin treatment

Mouse primary pancreatic cancer cell lines were cultured in DMEM containing 10% FBS and 100 IU/ml penicillin/streptomycin supplemented with glutamine. At 70% confluence, cells were treated with nutlin 2 µM/mL for 16 h then lysates were collected as described for immunoblotting below.

### Immunohistochemistry

For immunohistochemical analysis, sections were de-paraffinized, rehydrated, and boiled in a microwave for 10 min in either 10 mM citrate buffer (pH 6.0) or Tris-EDTA (pH 9.0) for antigen retrieval. Inactivation of endogenous peroxidase activity was performed by incubation with 3% hydrogen peroxide in methanol for 10 min followed by blocking with normal goat serum and then primary antibody incubation overnight at 4°C. Biotinylated secondary antibodies were applied for 30 min followed by Vectastain Elite ABC-HRCP reagent for 15 min and visualised with DAB (Vector Laboratories; SK-4100). Sections were counterstained with haematoxylin, dehydrated, and mounted for imaging.

### Immunoblotting

For immunoblotting, cells were lysed in urea buffer containing 8 M urea, 1 M thiourea, 0.5% CHAPS, 50 mM DTT, and 24 mM spermine. Protein extracts were loaded into SDS-polyacrylamide gels and transferred onto nitrocellulose membrane (Protran). Resulting blots were incubated with primary antibody for 16 h at 4°C, and then with the appropriate HRP-conjugated secondary antibody (Dako). Protein expression was visualised by enhanced chemiluminescent detection (Amersham Biosciences) using X-ray film (Fujifilm).

### Cytokine profiling

For cytokine profiling, pancreas tissue following CP was lysed in urea buffer. Tissue lysates were used with Mouse Cytokine Array Panel A (R&D Systems) following the manufacturer’s instructions. Membranes were exposed with X-ray film (Fujifilm) and scanned at an optimal exposure time.

### Sequencing p53

Total RNA was isolated from cells using the Qiagen RNeasy kit. The RNA was converted to cDNA using Super Script (Invitrogen) and the p53-specific primer 5′-GTG AGA TTT CAT TGT AGG TGC CAG G-3′. The cDNA mix was then used as a template to generate overlapping PCR fragments using Promega GoTaq G2 Green master mix and the following primers: Fragment 1: 5′ GTT GCT GGG ATT GGG ACT TTC C 3′ and 5′ GTA GCA TGG GCA TCC TTT AAC TC 3′; Fragment 2: 5′GCT ATT CTG CCA GCT GGT GAA GAC 3′ and 5′ GTC TGA GTC AGG CCC CAC TTT C 3′; Fragment 3: 5′GCT ATT CTG CCA GCT GGT GAA GAC 3′ and 5′-GTG AGA TTT CAT TGT AGG TGC CAG G-3′. PCR fragments were purified using the Qiagen Gel purification kit and sequenced using the following primers: 5′ CCC AGG ATG TTG AGG AGT TTT TTG 3′; 5′ GCA TGA ACC GCC GAC CTA TCC 3′; 5′ GTA GCA TGG GCA TCC TTT AAC TC 3′; 5′ GGG ACA AAA GAT GAC AGG G 3′.

### Fluorescence-activated cell sorting (FACS)

Pancreata following CP were placed in 5 mL collagenase buffer (125 U/mL collagenase IV (Worthington), 40 U/mL DNaseI (Roche), 25 mM HEPES, and 0.5% HI-FCS in 1X HBSS) in C tubes (Miltenyi Biotec). Tissues were dissociated using programme ‘m_imp_tumor_04’ on a gentleMACS dissociator (Miltenyi Biotec), incubated at 37 °C for 40 min with gentle agitation, then dissociation was repeated using programme ‘m_imp_tumor_04’. Following dissociation cells were filtered through a 100 µm cell strainer (Falcon). Spleens were processed by mechanical digestion on 70 μm strainers.

Red blood cells were lysed with ACK lysis buffer, and cells were stained for 30 min on ice with Zombie Green Viability Dye (BioLegend; 1:1000) or Zombie NIR Viability Dye (BioLegend; 1:1000) and directly conjugated antibodies in 0.5% HI-FCS and 0.05% sodium azide in PBS. The following surface antibodies were used: APC-Cy7 anti-CD3 (17A2; 1:100), PE-Cy5 anti-CD4 (RM4-5; 1:300), BV421 anti-CD4 (GK1.5; 1:100), FITC anti-CD4 (RM4-5, eBioscience; 1:100), APC anti-CD8α (53-6.7; 1:200), BV510 anti-CD8α (53-6.7; 1:100), BV785 anti-CD8α (53-6.7; 1:200), BV510 anti-CD11b (M1/70; 1:100), PerCP-Cy5.5 anti-CD11b (M1/70; 1:50), BV421 anti-CD11c (N418; 1:100), PE-Cy7 anti-CD11c (N418; 1:250), BV650 anti-CD19 (6D5; 1:150), BV510 anti-CD25 (PC61; 1:50), FITC anti-CD45 (30-F11; 1:100), PE/Dazzle 594 anti-CD49b (DX5; 1:50), PE-Cy7 anti-CD68 (FA-11; 1:250), BV421 anti-F4/80 (BM8; 1:50), BV711 anti-F4/80 (BM8; 1:100), BV785 anti-Ly6C (HK1.4; 1:500), PerCP-Cy5.5 anti-Ly6G (1A8; 1:250), Alexa Fluor 700 anti-MHCII (M5/114.15.2; 1:250), and PE anti-TCRγδ (UC7-13D5; 1:100). For intracellular staining, cells were fixed with Fixation/Permeabilization Buffer (eBioscience) overnight at 4 ^o^C, washed with Permeabilization Buffer (eBioscience), incubated with directly-conjugated antibodies in Permeabilization Buffer for 30 min on ice, washed, and acquired directly. The following intracellular antibodies were used: PE-Cy5 anti-FoxP3 (FJK-16s, eBioscience; 1:100). All antibodies were purchased from BioLegend unless otherwise indicated.

Following fixation, cells were read using 3–11 fluorophore flow cytometry on a LSRFortessa X-20 (BD Biosciences) with 405, 488, 561 and 633 lasers. Compensation matrices were determined for each individual acquisition date using unstained and single fluorophore-stained controls. Data was analysed using FlowJo software (Tree Star) and cell numbers were calculated from tissue cell counts preceding staining.

### RNA sequencing (RNA-seq)

Total RNA was extracted from cells with the RNeasy Mini Kit (QIAGEN) following the manufacturer’s protocol with on-column DNase I digestion performed during RNA extraction. Library preparation was performed using the QuantSeq FWD 3′ mRNA-Seq Library Prep Kit (Lexogen) according to manufacturer’s protocol. High output single read sequencing was conducted on the Illumina NextSeq500 platform according to manufacturer’s instructions.

RNA-seq data were analysed by RPO with reads aligned to GRCm38.ERCC genome. Gene counts were generated from the aligned reads using featureCounts and differential expression was conducted in R (v4.2.1) using DESeq2. Briefly, DEseq2 was used to identify DEGs with adjustment for mouse sex. Genes having an adjusted *P* < 0.1 were called as significant. Moderated log_2_ fold changes were used to rank genes for gene set enrichment analysis (GSEA). For visualisation with volcano plots, adaptive shrinkage was applied to log_2_ fold change values. For heatmaps, DEGs were ordered on log_2_ fold change and then plotted as regularised log transformed counts, where the effect of library size differences are removed. Comparison of overlapping genes was performed using Enrichr and the TRRUST reference database of transcription factor targets [56, 57].

### Histological and image analysis

To measure acinar-to-ductal metaplasia (ADM) ex vivo all acini and ducts were counted per well are reported as a percentage for at least two independent experiments per condition.

To quantify ADM in vivo H&E-stained whole tissue sections were first scanned with Hamamatsu NanoZoomer S210 (Hamamatsu, Japan) using 40× objective lens. Tissue sections from age-matched (12- to 15-week-old) KC (*n* = 5) and KC;iASPP^Δ8/Δ8^ (*n* = 4) were viewed using NDP.view2 (Hamamatsu, Japan) and five 1 mm^2^ areas per slide were counted for ADM events.

### Human TMA analysis

Expression of iASPP in human pancreatic disease was analysed using a tissue microarray (TMA) obtained from US Biomax (PA2081b). A weighted histoscore of pancreatic tissue was calculated from the sum of (1 × % weak staining) + (2 × % moderate staining) + (3 × % strong staining) of tissue stained with iASPP antibody (LXO49.3).

### Human survival data analysis

Gene expression, and clinical data were obtained via the TCGAbiolinks (2.24.3) R package for TCGA-PAAD, and GDC data portal (https://portal.gdc.cancer.gov/repository) for CPTAC-3 [33, 34]. Only pancreatic ductal adenocarcinoma (PDAC) samples were included for downstream analysis. To account for library size variations, raw read counts were transformed to counts-per-million (CPM) and log-CPM using the ‘edgeR’ Bioconductor package (3.38.0). Genes with low number of reads were filtered out using the ‘filterByExpr’ function and inter-sample variation was normalised using the trimmed mean of M-values (TMM) method of ‘edgeR’.

Tumour subtyping of normalized RNA data was performed based on the Eyres et al. method [58]. Gene signatures were obtained from the original publications of Moffitt et al., Collisson et al., and Bailey et al. [2, 59, 60]. ‘GSVA’ package (1.44.0) was then used to generate gene signature enrichment scores followed by K-means clustering using the ‘cluster’ (2.1.3) package. The optimal number of clusters (K values) was determined using the Elbow method. Based on the enrichment scores, samples were labelled ‘Basal’, and ‘Classical’ for Moffitt et al; ‘QMA’, ‘Classical’, and ‘Exocrine’ for Collisson et al; ‘Squamous’, ‘Progenitor’, ‘ADEX’, and ‘Immunogenic’ for Bailey et al. In addition, ‘Consensus’ basal-like and classical subtypes were assigned to samples enriched for Basal/QMA/Squamous and Classical (Moffitt)/Classical (Collisson)/Progenitor respectively.

Kaplan–Meier plots were performed using the ‘survival’ (3.3-1), and ‘survminer’ (0.4.9) R packages. Continuous PPP1R13L expression was transformed to categorical groups via the maximally selected rank statistics (maxstat) method of the ‘survival’ package. The log-rank test of the ‘survival’ package was used to statistically compare survival estimates between groups.

### Statistical analysis

Statistical significance between groups was calculated by two-tailed Student’s *t* test unless specified otherwise in the figure legend. No statistical method was used to predetermine sample size. Group allocation and outcome assessment were not performed in a blinded manner. Survival was measured using the Kaplan-Meier method. Where Student’s *t* test was performed, normality was tested using the D’Agostino and Pearson test with *p* = 0.05. The variance was compared using an F test and was similar between the groups being statistically compared. Statistical significance was calculated using GraphPad 8 software (GraphPad Software).

## Supplementary information

Figure S6C Western Blot - Bax p21 p53.2

Figure S6C Western Blot - iASPP

Figure S3A Western Blot - iASPP

Figure S3A Western Blot - p53

Figure S3A Western Blot - Beta Tubulin

Figure S6B Western Blot - Beta Tubulin

Figure S6B Western Blot - iASPP

Figure S6C Western Blot - Actin

Figure S6C Western Blot - Bax p21 p53.1

Supplemental Figure Legends

AJ Checklist

Figure S1A-K

Figure S2A-I

Figure S3A-B

Figure S4A-G

Figure S5

Figure S6A-C

Figure S7A-D

Figure S7E-G

Figure S7H-I

## Data Availability

Raw data, original IHC microscopy images and western blots have been deposited to Mendeley (Mendeley data: 10.17632/bsm3s864fv.1) and are publicly available as of the date of publication. RNA-seq data generated in this publication have been deposited in NCBI’s Gene Expression Omnibus (GEO) and are accessible through GEO Series accession number GSE226595. Any additional information required to reanalyse the data reported in this paper is available from the lead contacts (Professor Xin Lu) upon request (xin.lu@ludwig.ox.ac.uk).
