# Supplementary figures and images for: p53 inhibitor iASPP is an unexpected suppressor of KRAS and inflammation-driven pancreatic cancer
Source: Cell Death Differ. 2023 Jun 3;30(7):1619–35. doi: 10.1038/s41418-023-01168-3 (PMC10307949; doi:10.1038/s41418-023-01168-3)

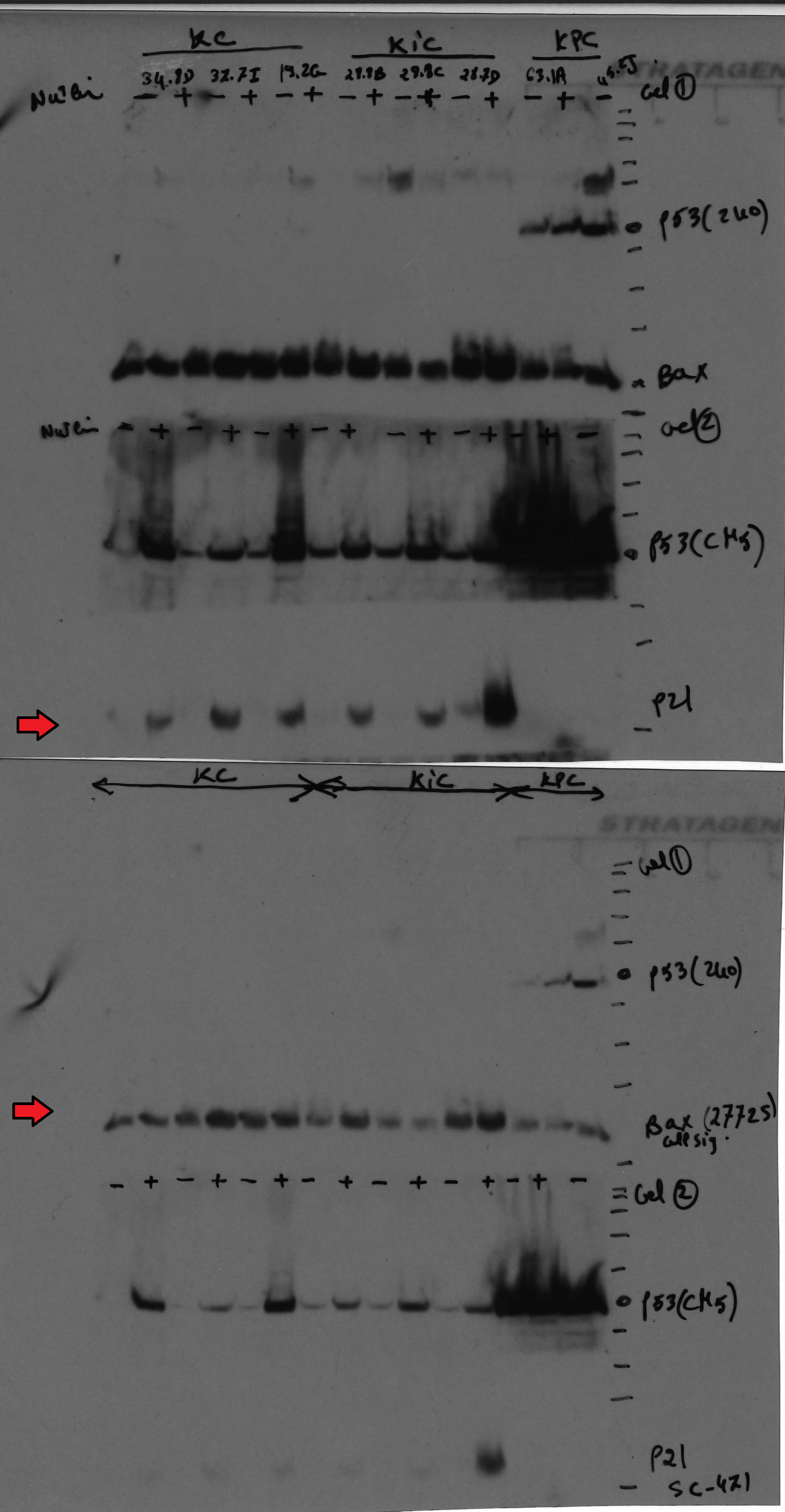

Supplement: Supplementary file 1 — Figure S6C Western Blot - Bax p21 p53.2 [file 41418_2023_1168_MOESM1_ESM.tif]

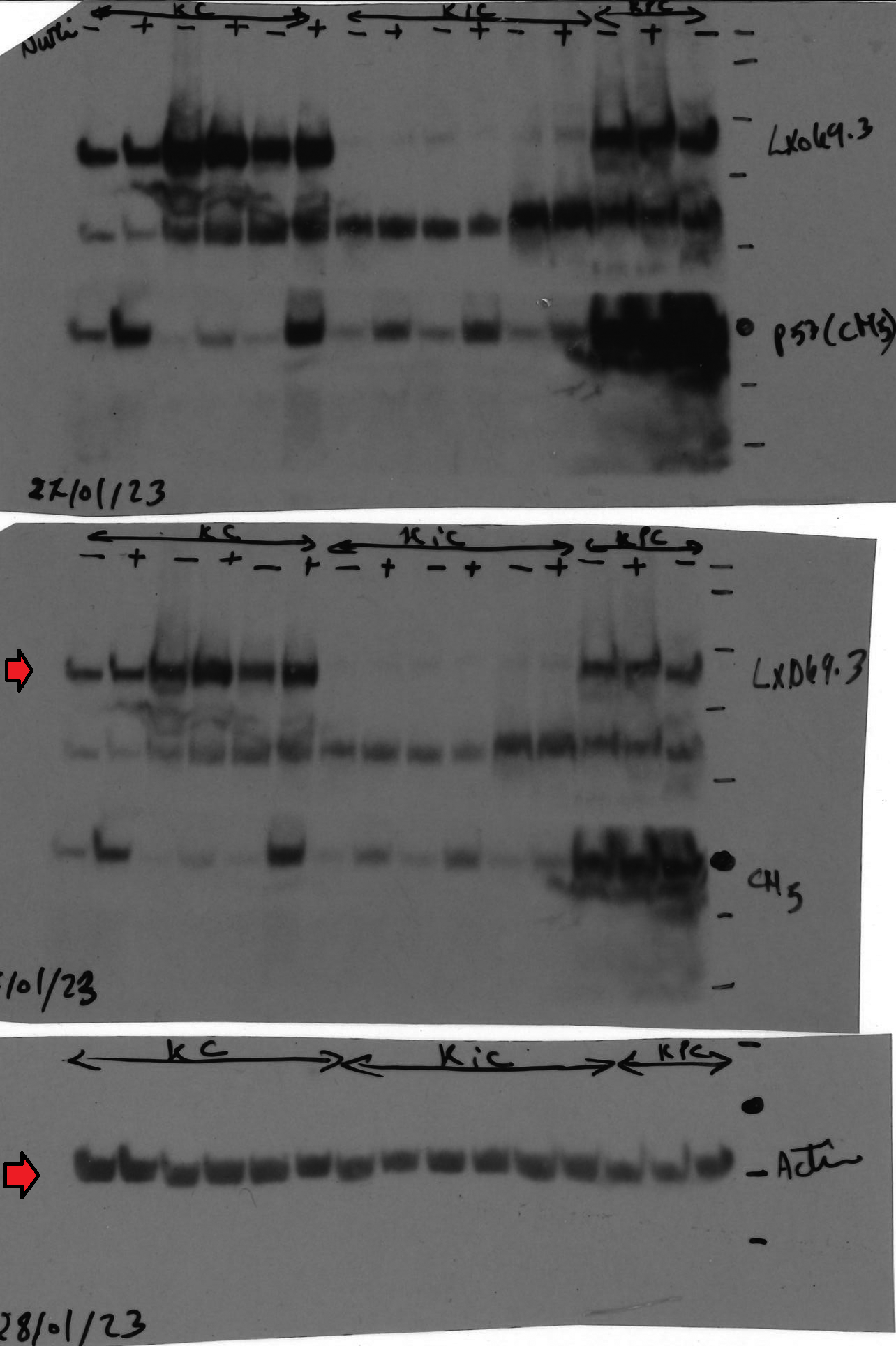

Supplement: Supplementary file 2 — Figure S6C Western Blot - iASPP [file 41418_2023_1168_MOESM2_ESM.tif]

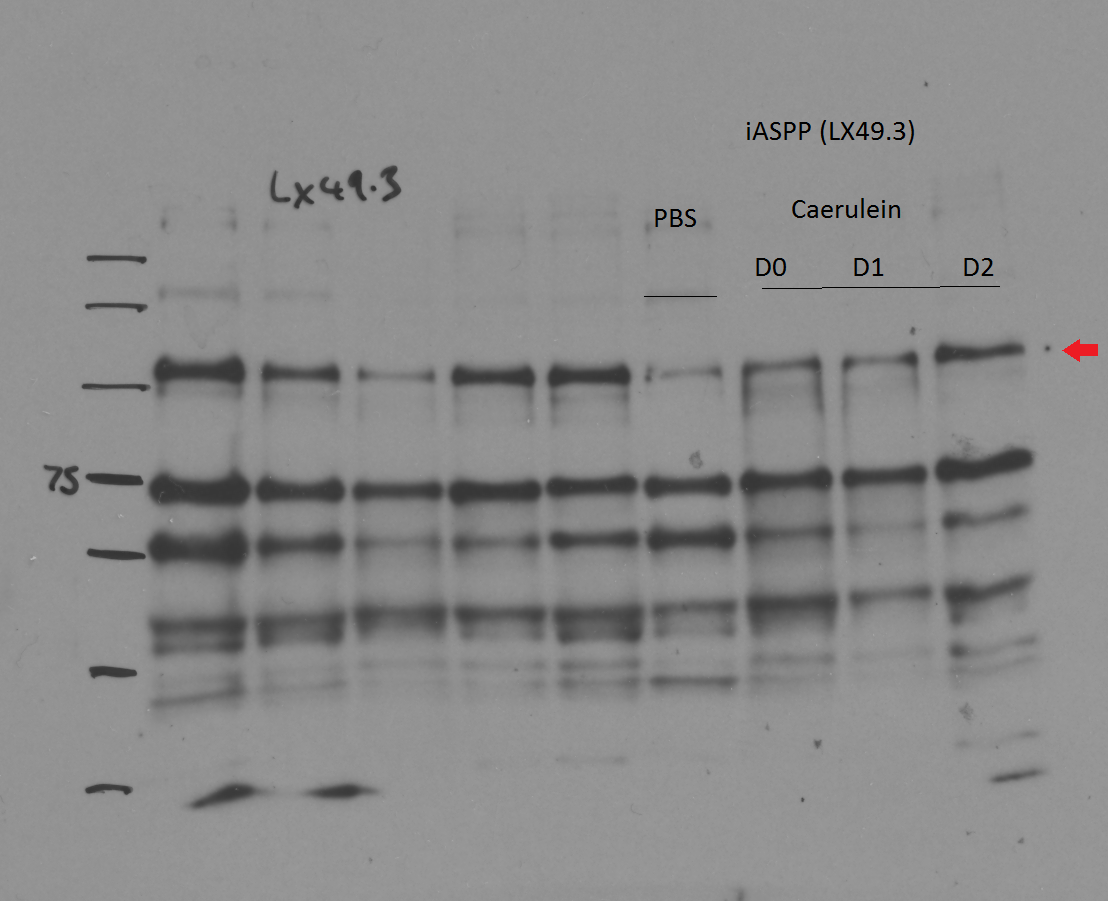

Supplement: Supplementary file 3 — Figure S3A Western Blot - iASPP [file 41418_2023_1168_MOESM3_ESM.tif]

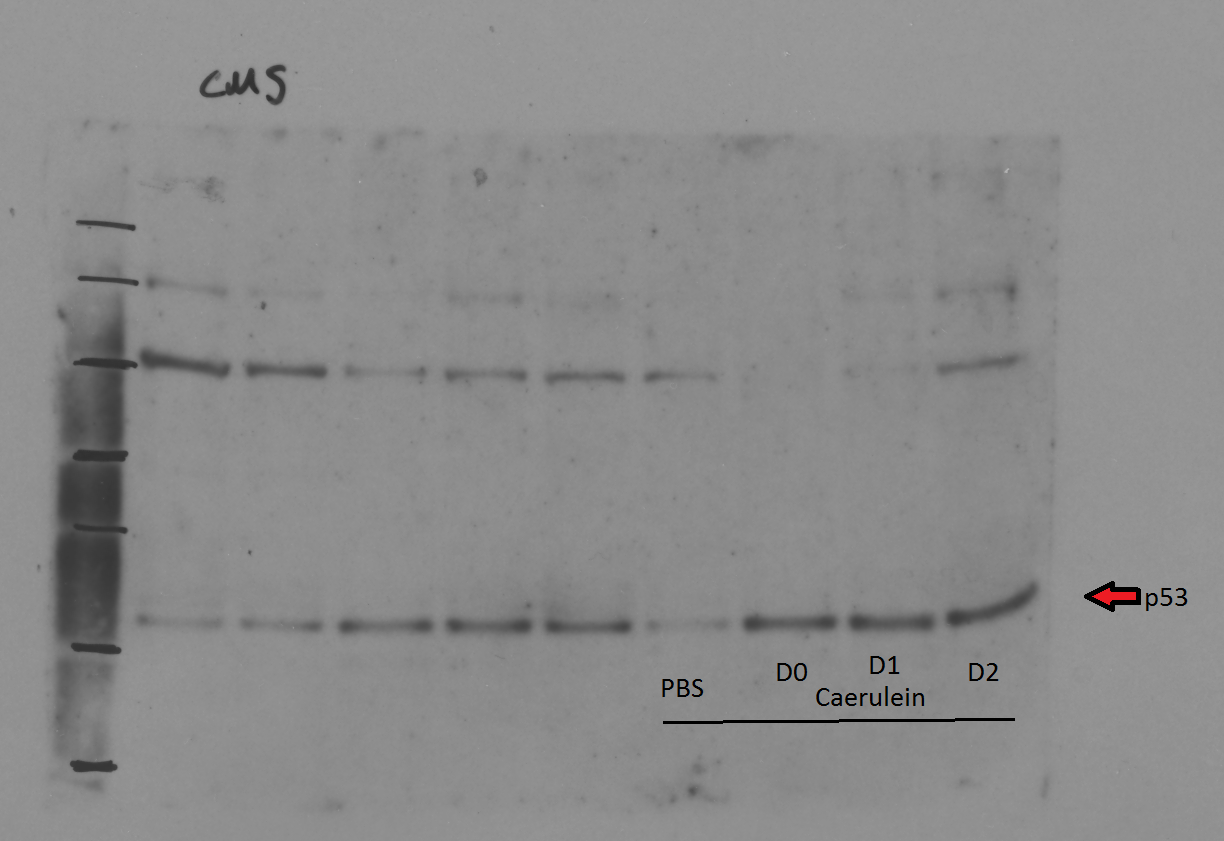

Supplement: Supplementary file 4 — Figure S3A Western Blot - p53 [file 41418_2023_1168_MOESM4_ESM.tif]

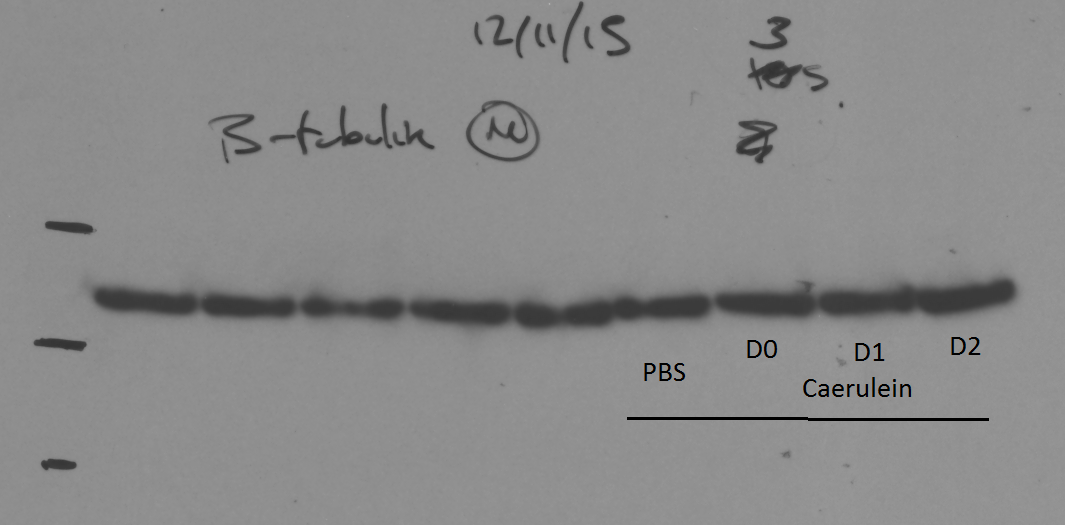

Supplement: Supplementary file 5 — Figure S3A Western Blot - Beta Tubulin [file 41418_2023_1168_MOESM5_ESM.tif]

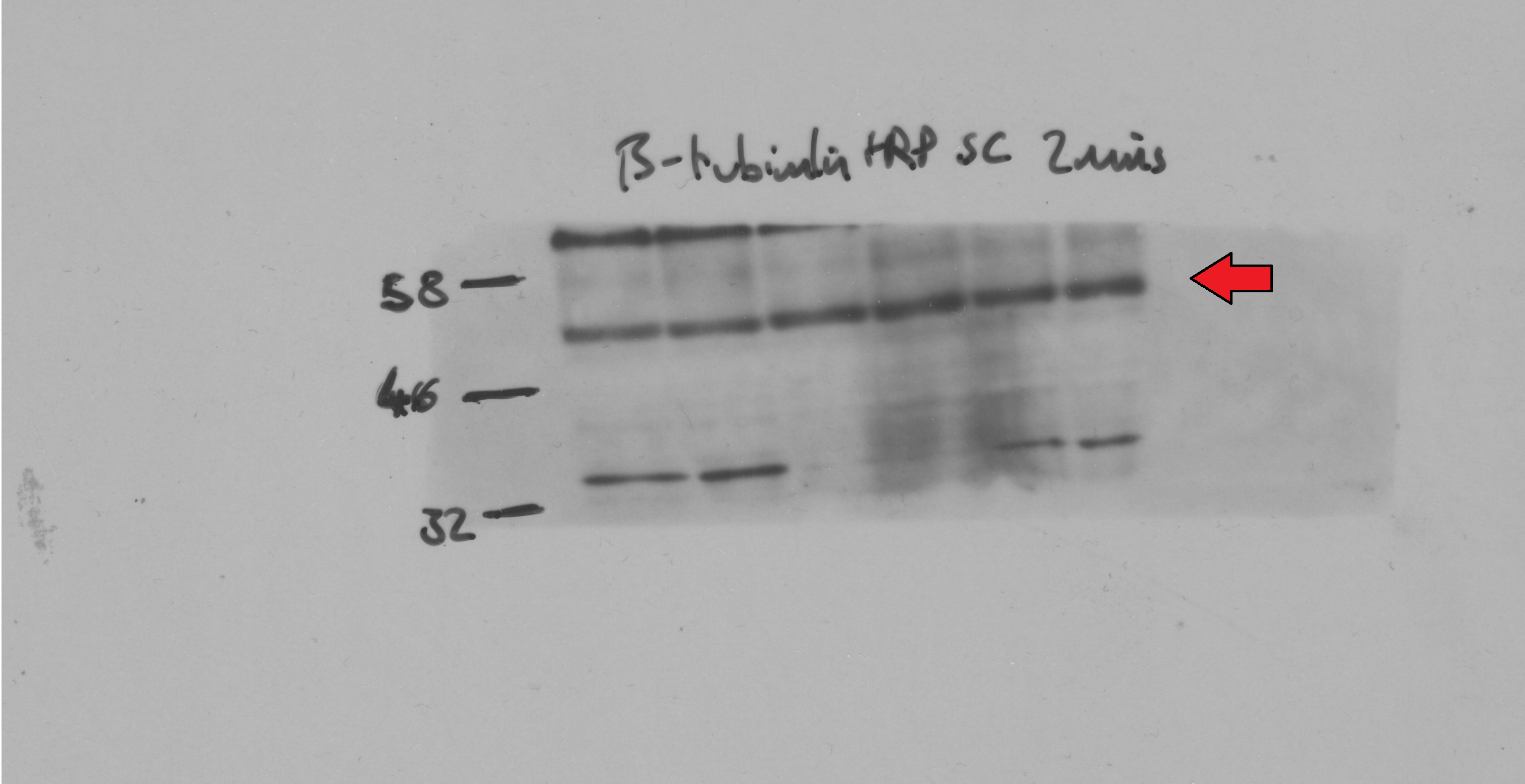

Supplement: Supplementary file 6 — Figure S6B Western Blot - Beta Tubulin [file 41418_2023_1168_MOESM6_ESM.tif]

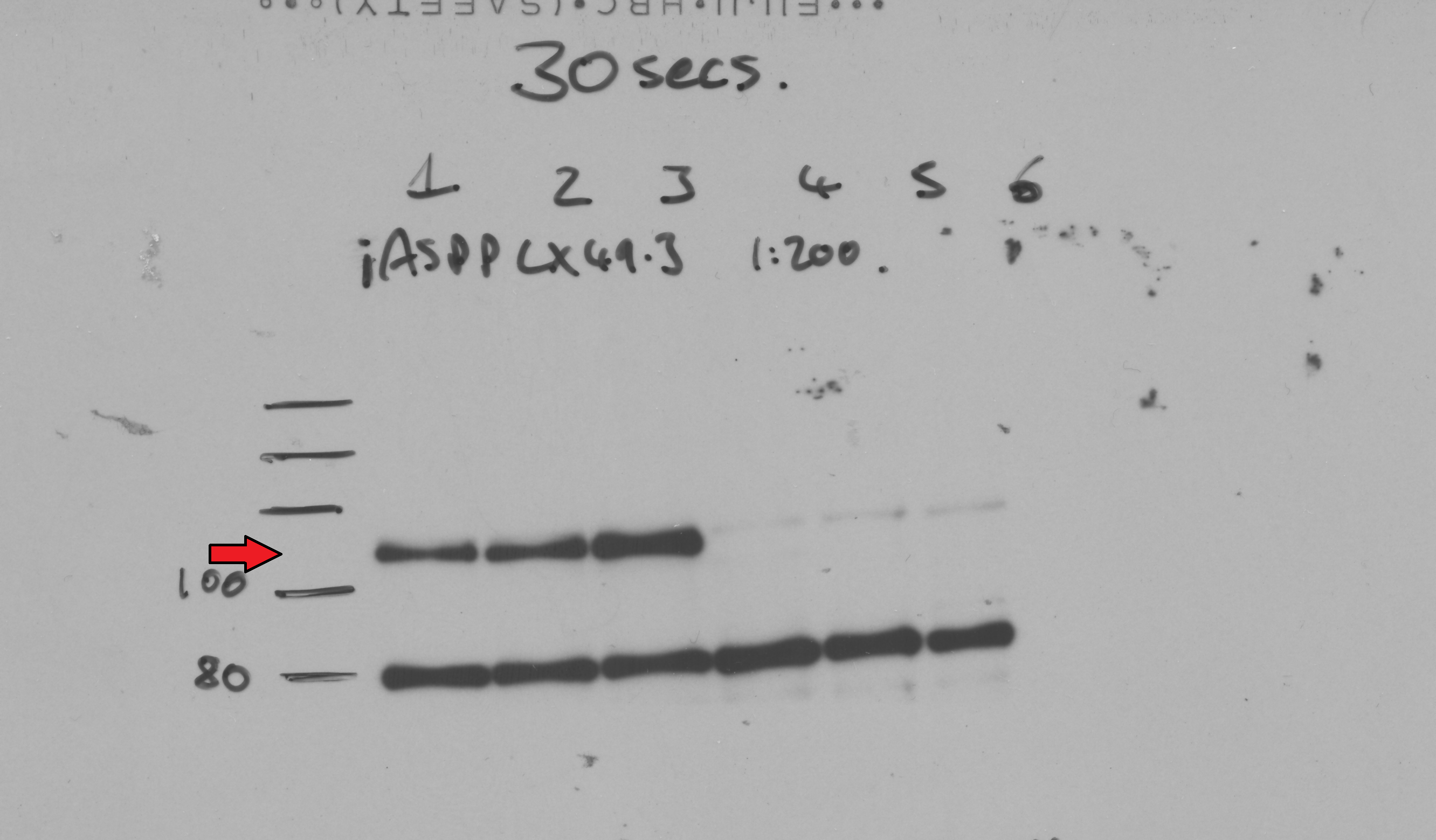

Supplement: Supplementary file 7 — Figure S6B Western Blot - iASPP [file 41418_2023_1168_MOESM7_ESM.tif]

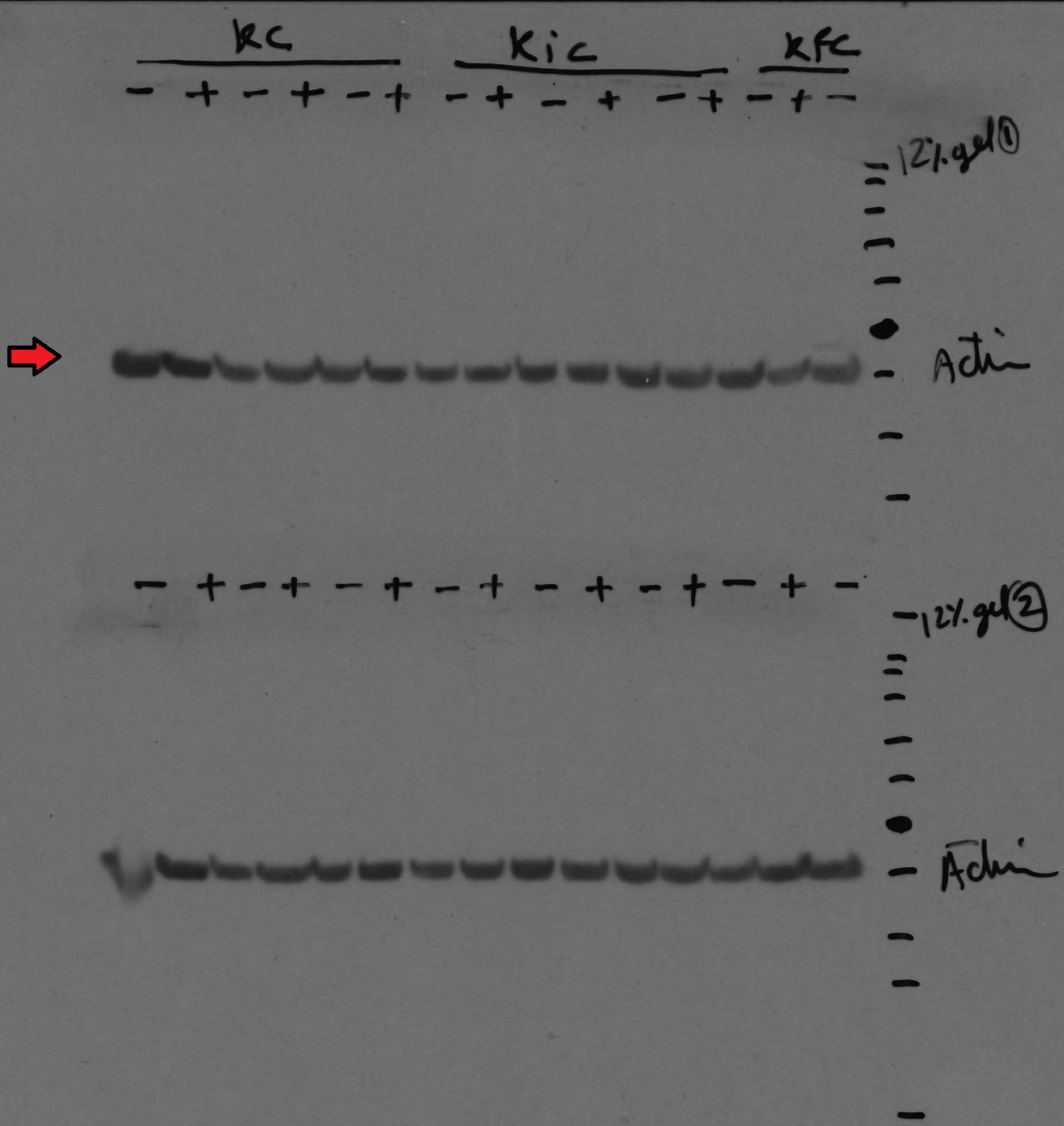

Supplement: Supplementary file 8 — Figure S6C Western Blot - Actin [file 41418_2023_1168_MOESM8_ESM.tif]

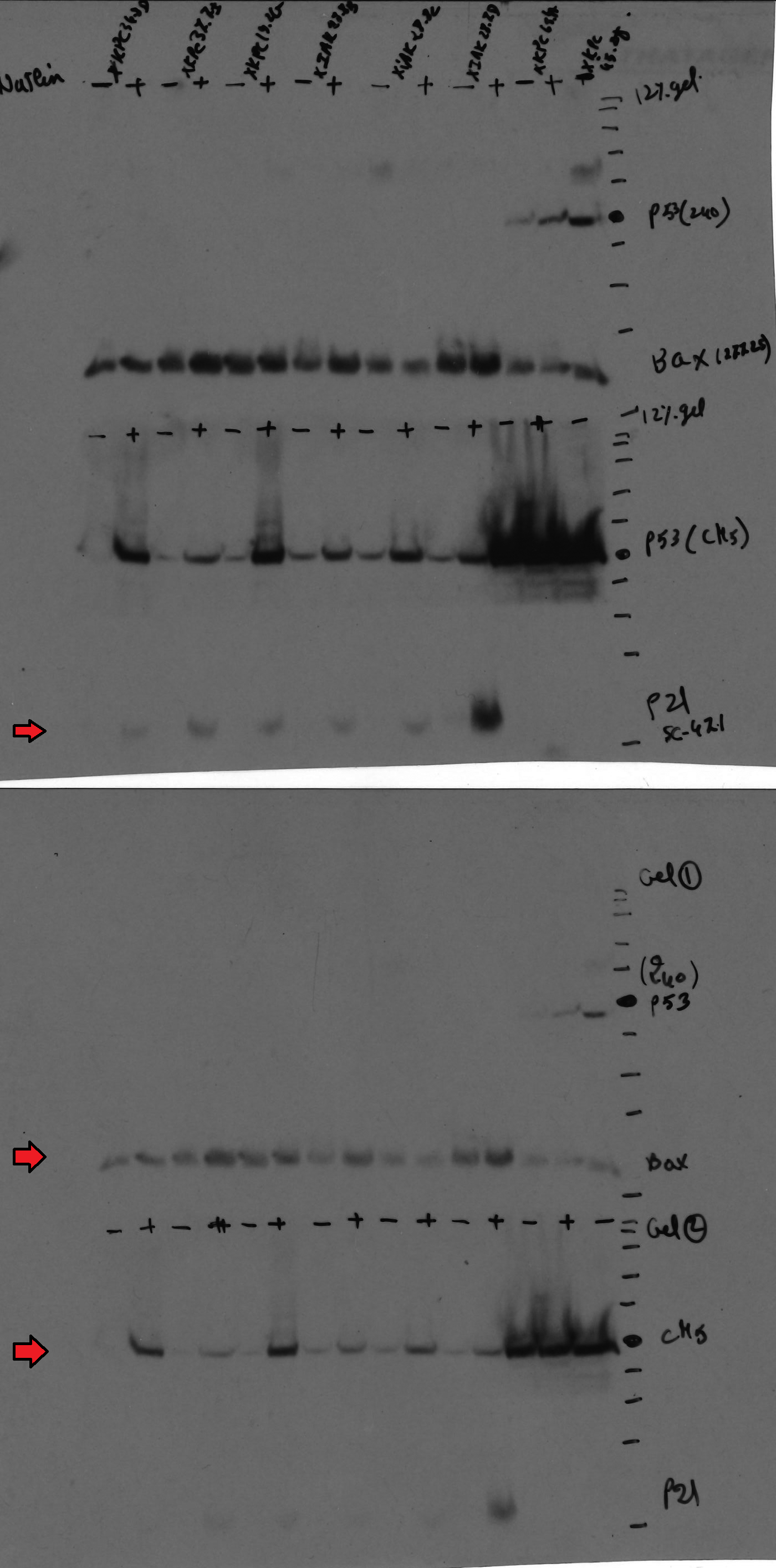

Supplement: Supplementary file 9 — Figure S6C Western Blot - Bax p21 p53.1 [file 41418_2023_1168_MOESM9_ESM.tif]

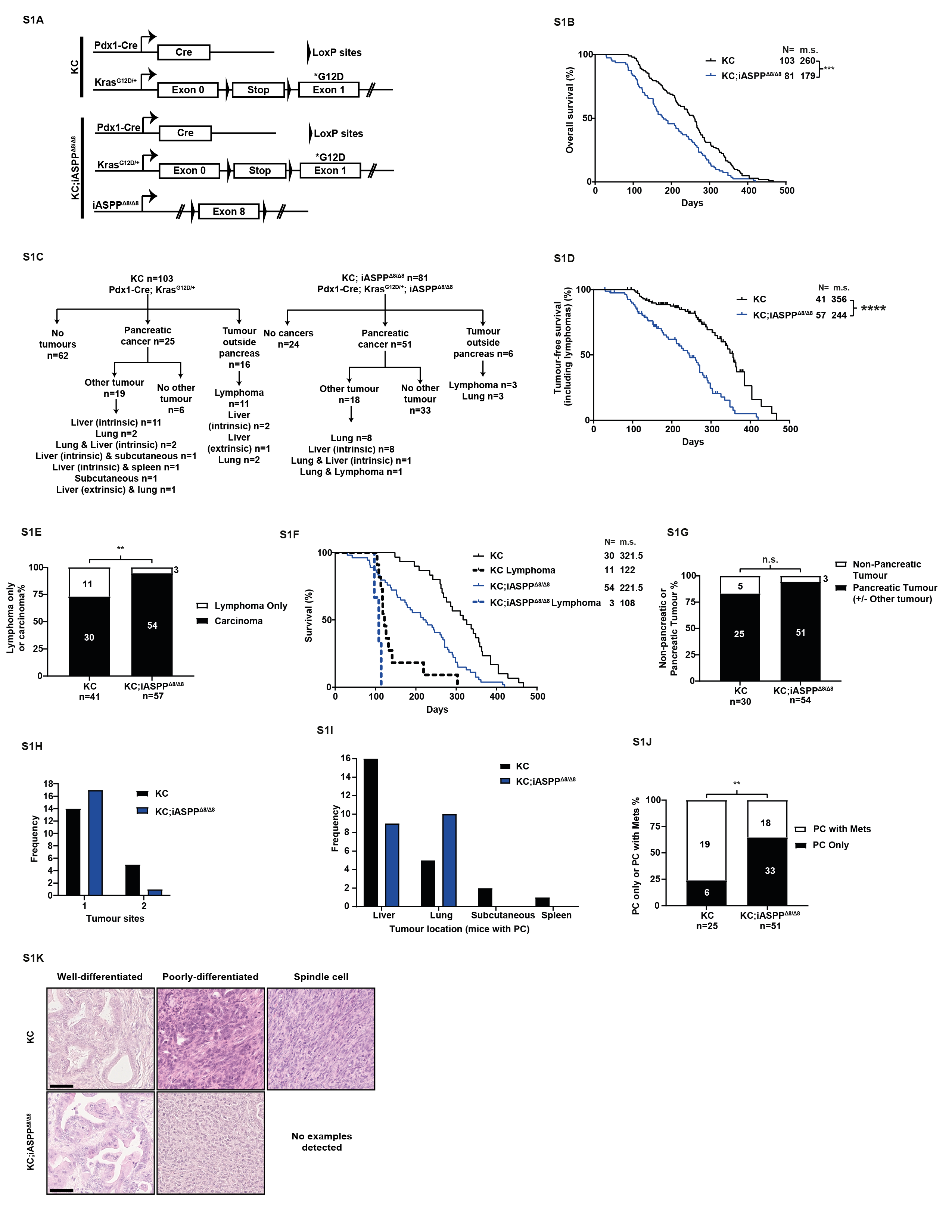

Supplement: Supplementary file 12 — Figure S1A-K [file 41418_2023_1168_MOESM12_ESM.png]

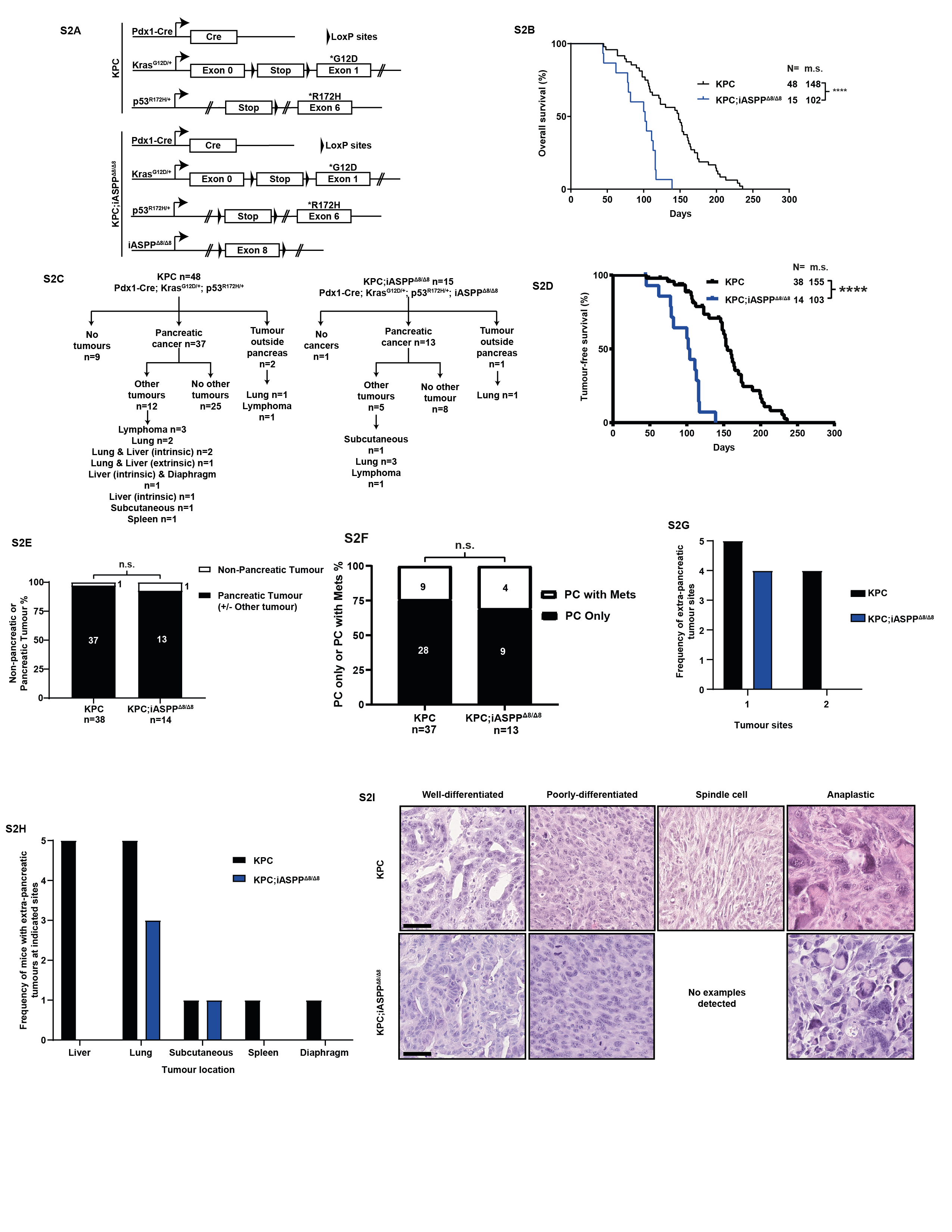

Supplement: Supplementary file 13 — Figure S2A-I [file 41418_2023_1168_MOESM13_ESM.png]

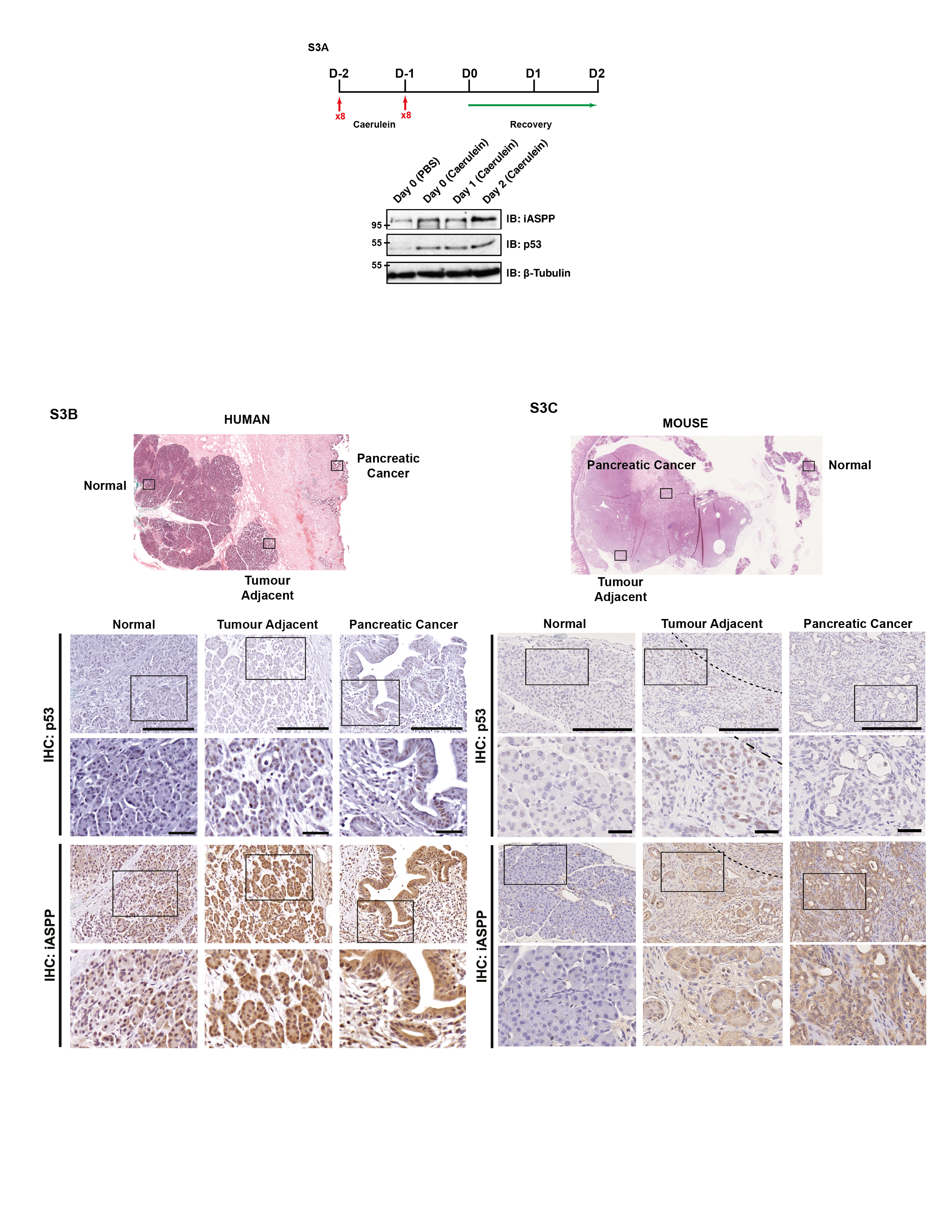

Supplement: Supplementary file 14 — Figure S3A-B [file 41418_2023_1168_MOESM14_ESM.png]

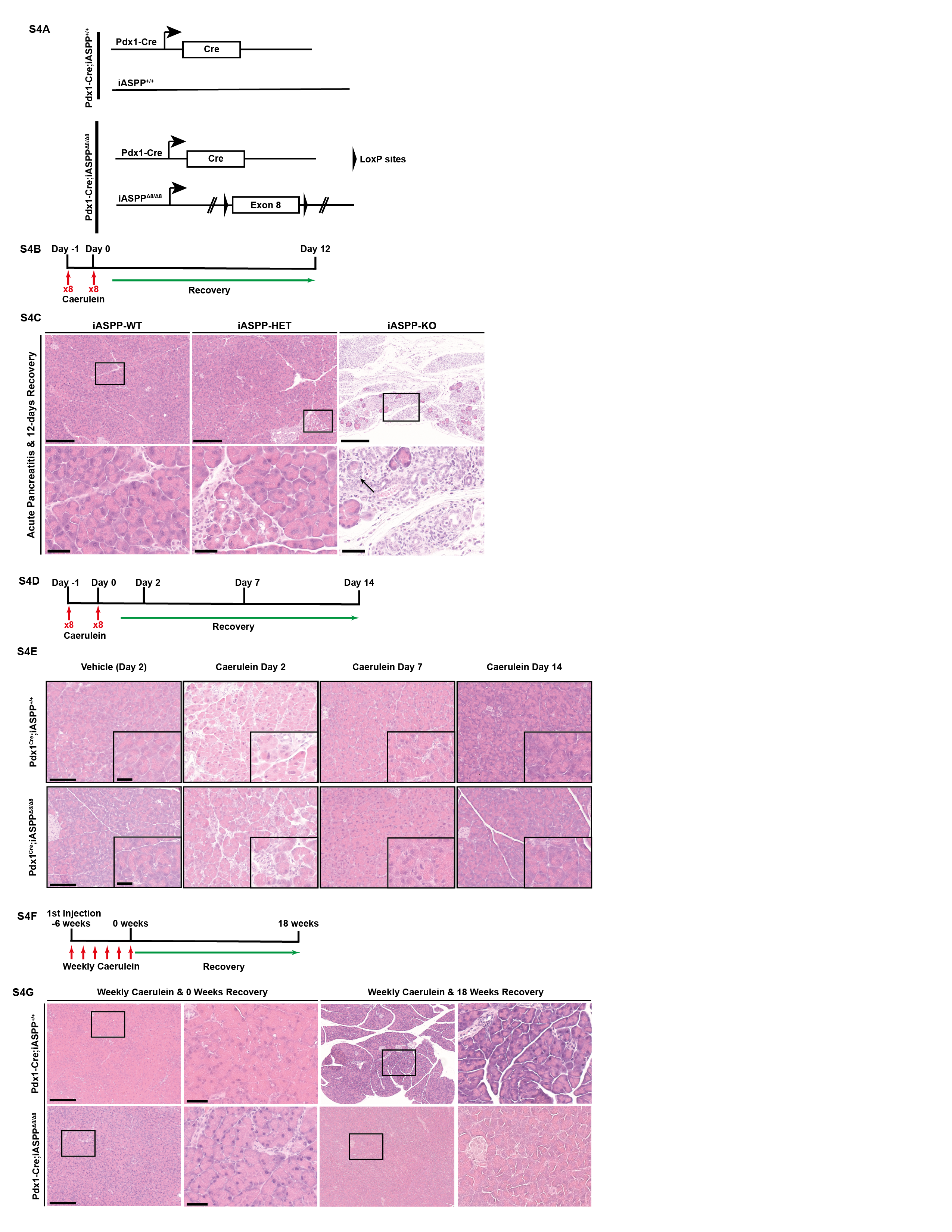

Supplement: Supplementary file 15 — Figure S4A-G [file 41418_2023_1168_MOESM15_ESM.png]

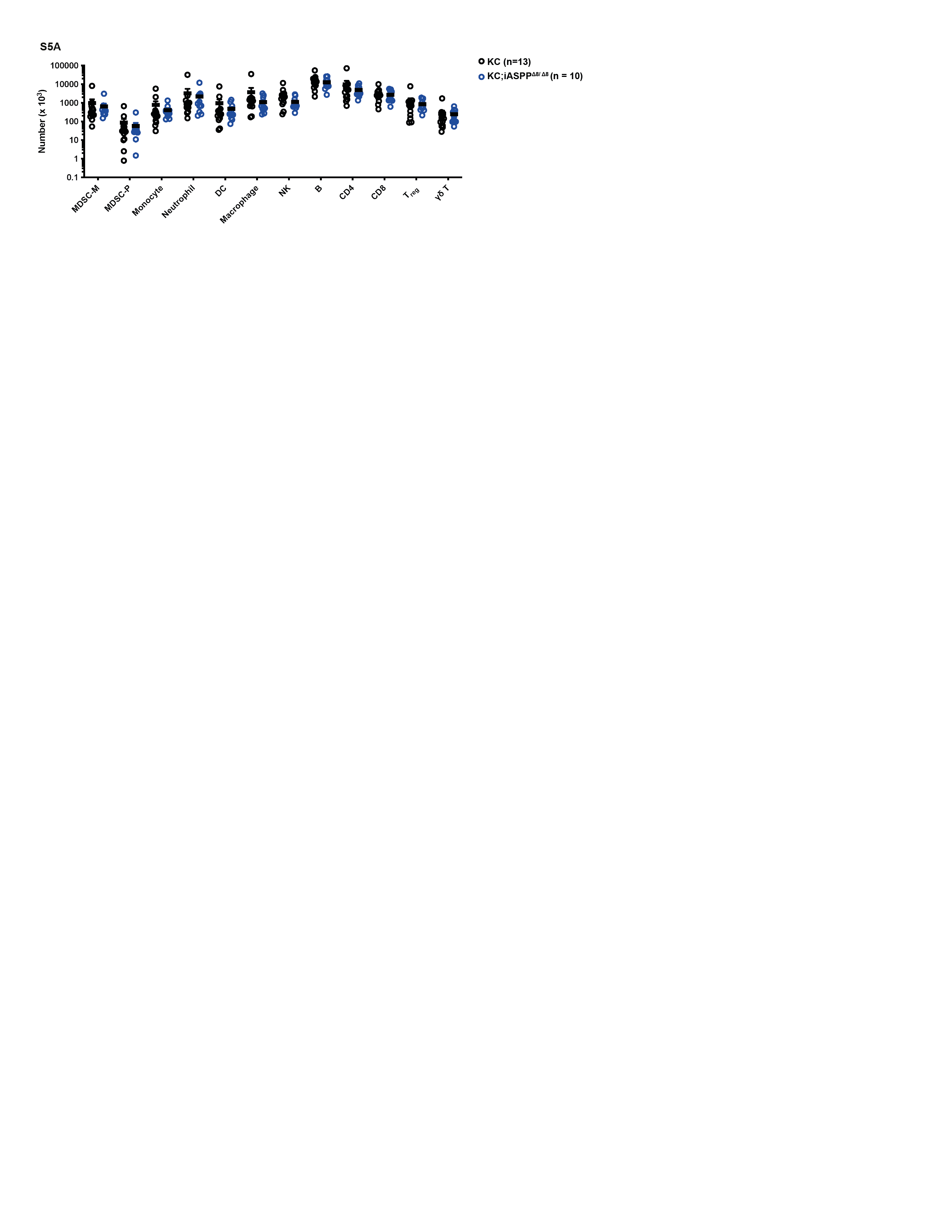

Supplement: Supplementary file 16 — Figure S5 [file 41418_2023_1168_MOESM16_ESM.png]

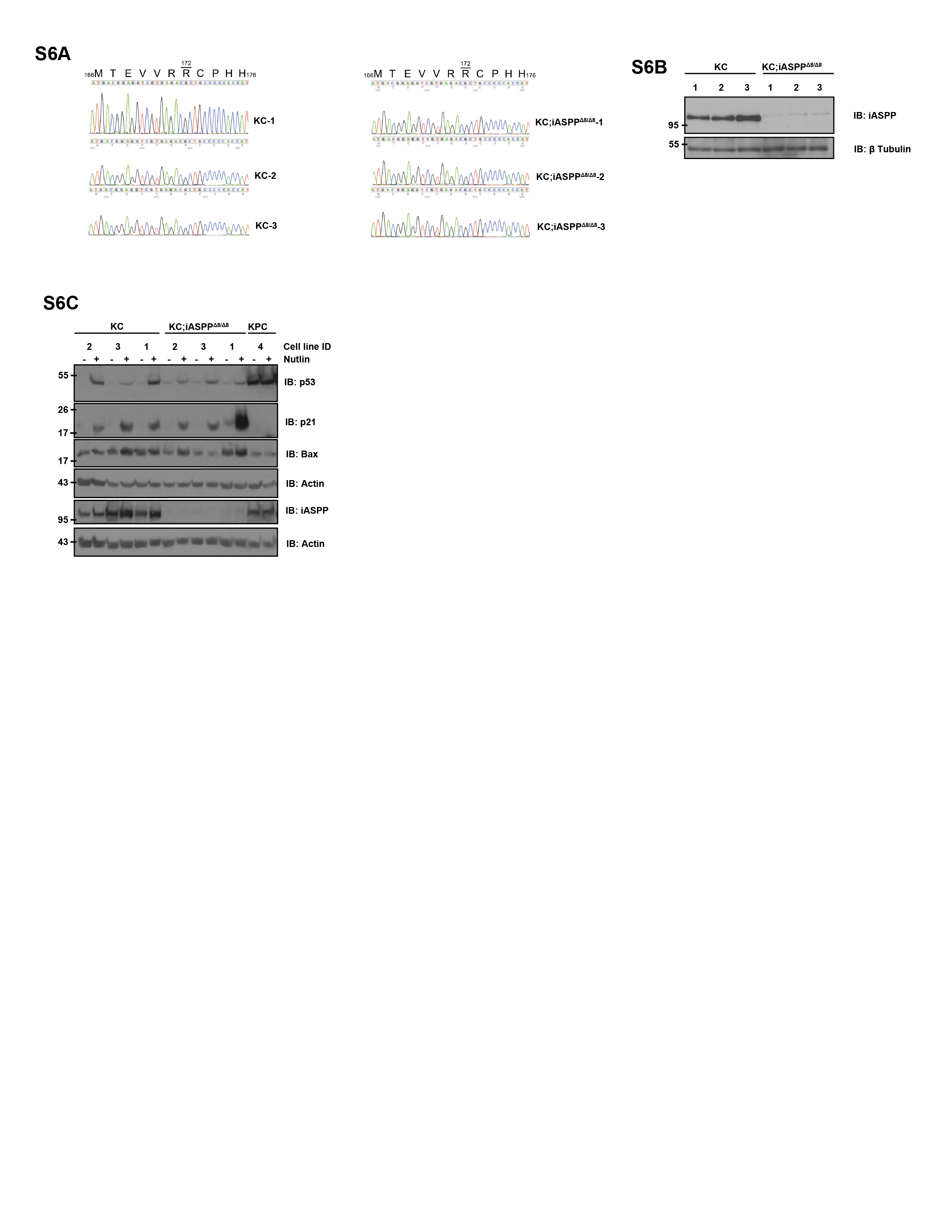

Supplement: Supplementary file 17 — Figure S6A-C [file 41418_2023_1168_MOESM17_ESM.png]

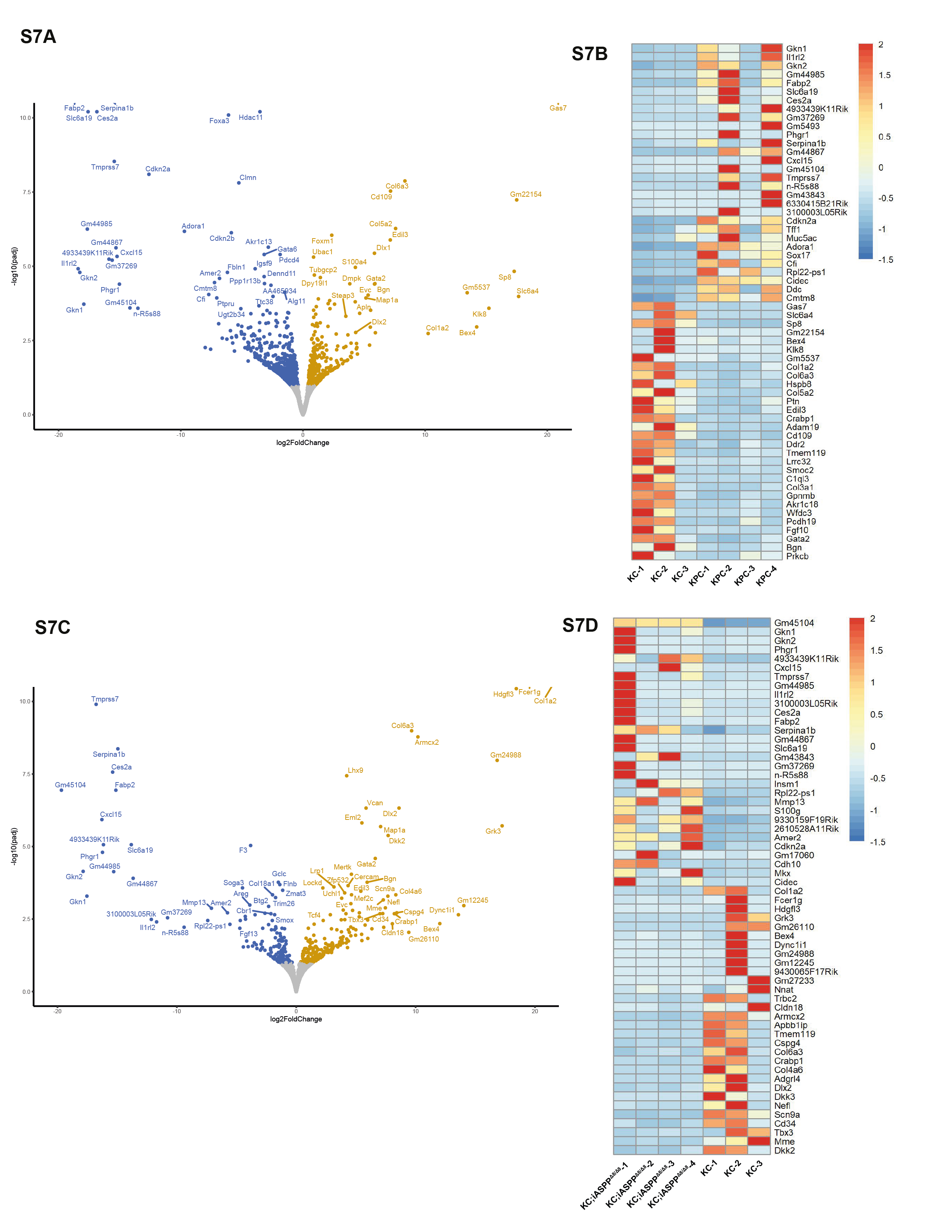

Supplement: Supplementary file 18 — Figure S7A-D [file 41418_2023_1168_MOESM18_ESM.png]

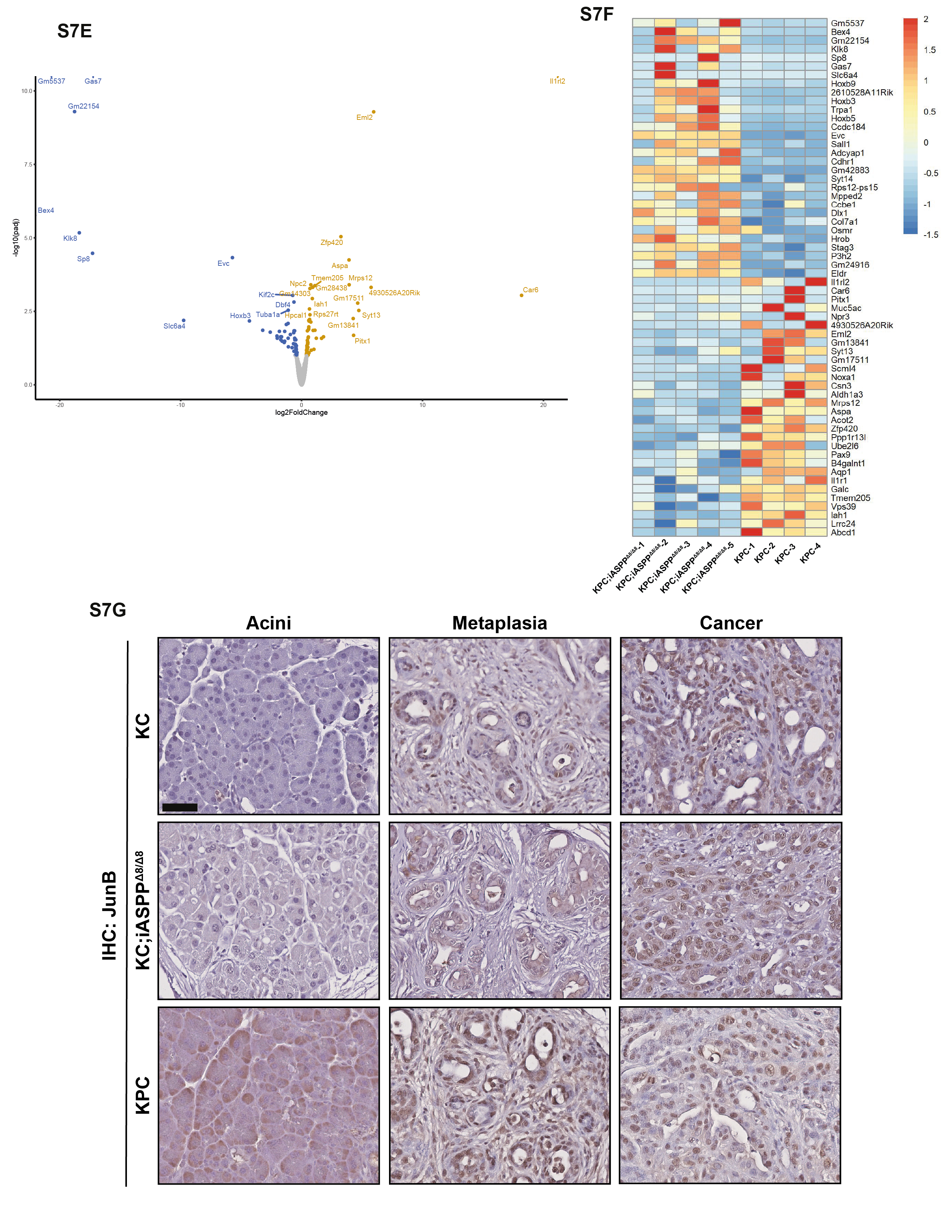

Supplement: Supplementary file 19 — Figure S7E-G [file 41418_2023_1168_MOESM19_ESM.png]

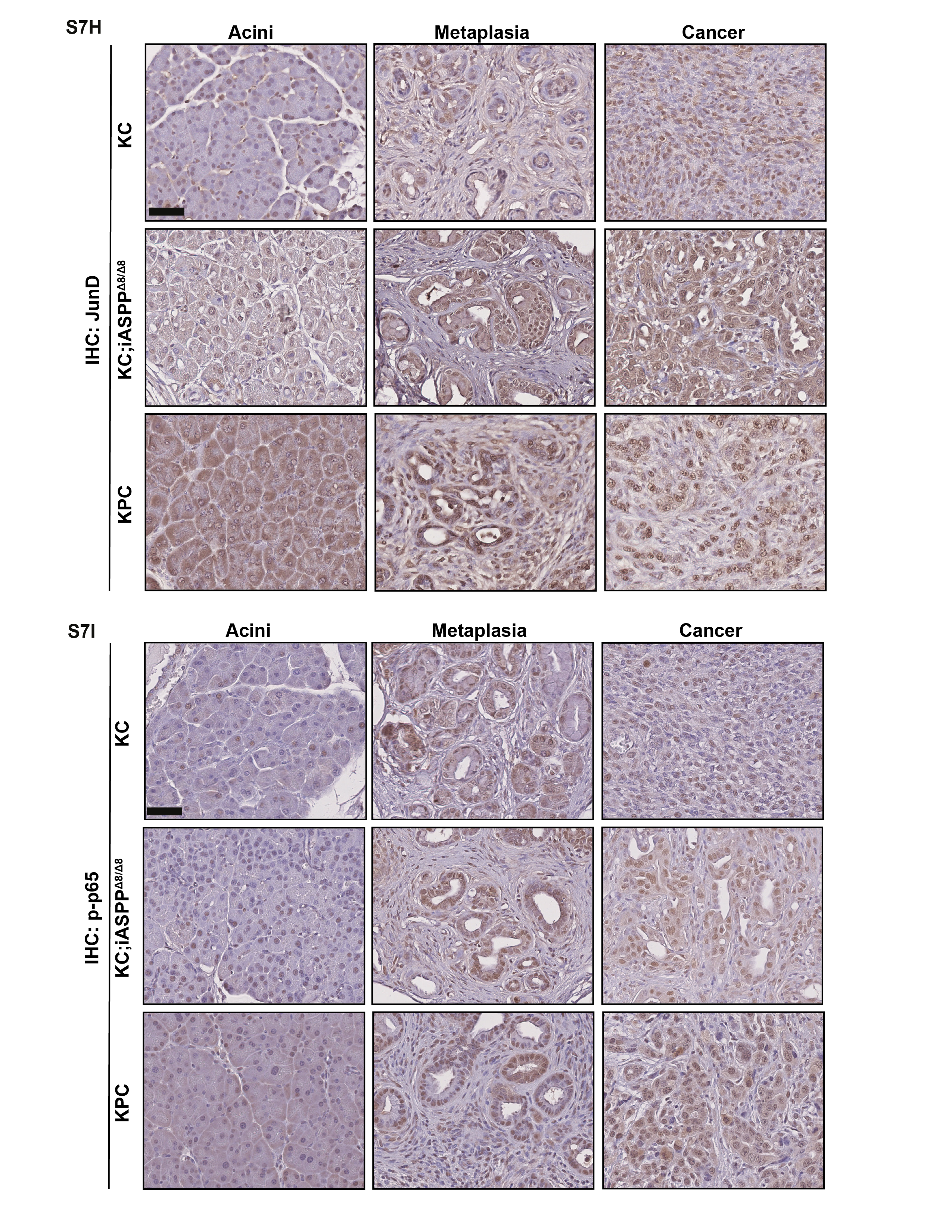

Supplement: Supplementary file 20 — Figure S7H-I [file 41418_2023_1168_MOESM20_ESM.png]
