# Supplementary material for: p53 inhibitor iASPP is an unexpected suppressor of KRAS and inflammation-driven pancreatic cancer
Source: Cell Death Differ. 2023 Jun 3;30(7):1619–35. doi: 10.1038/s41418-023-01168-3 (PMC10307949; doi:10.1038/s41418-023-01168-3)
Supplement: Supplementary file 10 — Supplemental Figure Legends [file 41418_2023_1168_MOESM10_ESM.docx]

## **p53 inhibitor iASPP is an unexpected suppressor of KRAS and inflammation driven pancreatic cancer**

Running title: p53 inhibitor iASPP as a pancreatic cancer suppressor

Paul Miller^1*^, Elliot H Akama-Garren^1^, Richard P. Owen^1^, Constantinos Demetriou^3^, Thomas M. Carroll^1^, Elizabeth Slee^1^, Khatoun Al Moussawi^1^, Michael Ellis^1^, Robert Goldin^2^, Eric O’Neill^3^, Xin Lu^1,4,*^

**Figure S1, related to Figure 1. iASPP unexpectedly suppresses oncogenic KRAS-driven PC onset**

(A) Genetic schematic of KC and KC;iASPP^Δ8/Δ8^ mice.

(B) Kaplan-Meier overall survival of KC and KC;iASPP^Δ8/Δ8^ mice. *** = p <0.001; n.s., not significant, log-rank test. N=, number of mice; m.s., median survival.

(C) Tumour spectrum in the KC and KC;iASPP^Δ8/Δ8^ cohorts.

(D) Kaplan-Meier tumour-free survival including lymphomas of KC and KC;iASPP^Δ8/Δ8^ mice in the overall survival cohort. Number of mice and median survival reported for tumour-bearing mice. Non-tumour bearing mice censored. **** = p <0.0001; log-rank test. N=, number of mice; m.s., median survival.

(E) Percentage of mice with evidence of either carcinoma or lymphoma from the KC and KC;iASPP^Δ8/Δ8^ tumour-bearing cohorts. ** = p <0.01; n.s., not significant; Fisher’s exact test.

(F) Kaplan-Meier carcinoma- and lymphoma-free survival of KC and KC;iASPP^Δ8/Δ8^ mice in tumour-bearing cohort. Only mice with either a non-lymphoma tumour or lymphoma contributed to median survival (no censored mice). N=, number of mice; m.s., median survival.

(G) Percentage of mice with tumour outside the pancreas in the absence of PC or presence of PC (± other tumour) in the KC and KC;iASPP^Δ8/Δ8^, cohorts. n.s., not significant; Fisher’s exact test.

(H) Frequency of tumour sites outside the pancreas for KC and KC;iASPP^Δ8/Δ8^ mice in the survival cohorts with extra-pancreatic tumours.

(I) Frequencies of extra-pancreatic tumours in the KC and KC;iASPP^Δ8/Δ8^ survival cohort.

(J) Percentage of mice with primary pancreatic cancer only or pancreatic cancer with metastases in the KC and KC;iASPP^Δ8/Δ8^ pancreatic cancer-bearing mice. ** = p <0.01; Fisher’s exact test.

(K) Representative H&E sections of well-differentiated, poorly-differentiated, and spindle cell pancreatic cancers across the KC and KC;iASPP^Δ8/Δ8^ survival cohort. Scale bar, 50 µm.

**Figure S2, related to Figure 2. iASPP suppresses oncogenic KRAS-driven PC independently of p53**

(A) Genetic schematic of KPC and KPC;iASPP^Δ8/Δ8^ mice.

(B) Kaplan-Meier overall survival of KPC and KPC;iASPP^Δ8/Δ8^ mice. **** = p <0.0001; log-rank test. N=, number of mice; m.s., median survival.

(C) Tumour spectrum in KPC and KPC;iASPP^Δ8/Δ8^ mice.

(D) Kaplan-Meier tumour-free survival, excluding lymphomas, of KPC and KPC;iASPP^Δ8/Δ8^, mice in the overall survival cohort. Number of mice and median survival for tumour-bearing mice with non-tumour bearing and lymphoma-bearing mice censored. **** = p <0.0001, log-rank test. N=, number of mice; m.s., median survival.

(E) Percentage of mice with tumour outside the pancreas in the absence of PC or presence of PC (± other tumour) in the KPC and KPC;iASPP^Δ8/Δ8^, cohorts. n.s., not significant; Fisher’s exact test.

(E) Kaplan-Meier pancreatic cancer free survival of the KPC and KPC;iASPP^Δ8/Δ8^ mice in the tumour-bearing cohort. **** = p <0.0001; log-rank test. N=, number of mice; m.s., median survival.

(F) Percentage of mice with PC only and PC with additional extra-pancreatic tumour (excluding lymphoma) in the KPC and KPC;iASPP^Δ8/Δ8^ PC-bearing mice. n.s., not significant; Fisher’s exact test.

(G) Frequencies of extra-pancreatic tumours in the KPC and KPC;iASPP^Δ8/Δ8^ survival cohort.

(H) Frequency of tumour sites outside the pancreas for KPC and KPC;iASPP^Δ8/Δ8^ mice in the survival cohorts with extra-pancreatic tumours.

(I) Representative H&E sections of well-differentiated, poorly-differentiated, spindle cell, and anaplastic pancreatic cancers across the KPC, and KPC;iASPP^Δ8/Δ8^ survival cohort. Scale bar, 50 µm.

**Figure S3, related to Figure 3. Inflammation induces iASPP expression in vivo and iASPP mRNA level associates with good prognosis in human classical PC**

(A) Injection schedule for acute pancreatitis induction (Upper image). Western blot of iASPP and p53 following acute pancreatitis with caerulin and 2-days PBS injection (Lower image).

(B) Human pancreatic cancer sample depicting normal pancreas, tumour adjacent and pancreatic cancer (H&E, upper image). Immunohistochemical staining for p53 and iASPP in corresponding normal, tumour adjacent, and pancreatic cancer Scale bars, 100 µm in upper image, 40 µm in lower image.

(C) Mouse pancreatic cancer sample from KC mouse depicting normal pancreas, tumour adjacent and pancreatic cancer (H&E, upper image). Immunohistochemical staining for p53 and iASPP in corresponding normal, tumour adjacent (dashed line depicts tumour border), and pancreatic cancer. Scale bars, 100 µm in upper image, 40 µm in lower image.

**Figure S4, related to Figure 4. iASPP is a paradoxical suppressor of oncogenic KRAS- and inflammation-induced ADM in vivo**

(A) Genetic schematic of Pdx1-Cre;iASPP+/+ and Pdx1-Cre;iASPP^Δ8/Δ8^ mice.

(B) Injection schedule for induction of acute pancreatitis with hourly caerulein (50 µg/Kg) for 8 injections followed by culling after 12-days recovery.

(C) Representative H&E images of iASPP-WT (wild-type), iASPP-HET (heterozygous) and iASPP-KO (knockout) pancreata following acute caerulein and 12-days recovery. Upper image inset at each timepoint is below respective pancreas section. Black arrow indicates ADM in progress. Scale bars, 250 µm (upper images) and 50 µm (lower images).

(D) Injection schedule for induction of acute pancreatitis with hourly caerulein (50 µg/Kg) for 8 injections over 2-days followed by culling after 2-, 7-, and 14-day recovery.

(E) Representative H&E images of wild-type and Pdx1-Cre;iASPP^Δ8/Δ8^ pancreas following 2-days acute pancreatitis and 2-, 7- and 14-day recovery. Control mouse injected with vehicle and culled after 2-days. Scale bar, 100 µm and 50 µm (inset).

(F) Injection schedule of weekly caerulein (250 µg/Kg) over 6 weeks followed by planned culling at 0- and 18-weeks recovery.

(G) Representative H&E images of Pdx1-Cre; iASPP+/+ and Pdx1-Cre;iASPP^Δ8/Δ8^ pancreas following weekly caerulein after 0- and 18-weeks recovery. Upper image inset at each timepoint is below respective pancreas section. Scale bars, 250 µm (upper images) and 50 µm (lower images).

**Figure S5, related to Figure 5. iASPP regulates its surrounding immune microenvironment in response to pancreatitis and oncogenic KRAS**

(A) Immune cell profiling of KC (n=13) and KC;iASPP^Δ8/Δ8^ (n=10) spleen tissue samples following six weekly doses of caerulein determined by flow cytometry.

**Figure S6, related to Figure 6. iASPP suppresses PC via cell intrinsic and extrinsic pathways**

(A) Sequencing of TP53 gene in KC-1, KC-2, KC-3, KC;iASPP^Δ8/Δ8^-1, KC;iASPP^Δ8/Δ8^-2, KC;iASPP^Δ8/Δ8^-3 cell lines confirming absence of R172H mutation.

(B) Western blot of KC and KC;iASPP^Δ8/Δ8^ cell lines confirming reduced iASPP protein levels.

(C) Western blot of KC and KC;iASPP^Δ8/Δ8^ cell lines confirming induction of p53 with nutlin treatment along with p53-target gene expression with p21 and Bax. KPC cell line used as positive control for p53 expression and negative control for p21 and Bax induction in response to nutlin.

**Figure S7, related to Figure 7. iASPP deletion or p53 mutation on a KC background induces profound transcriptional overlap centred on inflammatory genes**

(A) Volcano plot for KC and KPC DEGs

(B) Heatmap of top 30 up- and downregulated genes for KC and KPC cell lines

(C) Volcano plot for KC and KC;iASPP^Δ8/Δ8^ DEGs

(D) Heatmap of top 30 up- and downregulated genes for KC and KC;iASPP^Δ8/Δ8^ cell lines

(E) Volcano plot for KPC and KPC;iASPP^Δ8/Δ8^ DEGs

(F) Heatmap of top 30 up- and downregulated genes for KPC and KPC;iASPP^Δ8/Δ8^ cell lines

(G) Representative immunohistochemical staining for JunB in acini, metaplasia and cancer examples for KC (n=3), KC;iASPP^Δ8/Δ8^ (n=3) and KPC (n=3) pancreata. Scale bar, 50 µm.

(H) Representative immunohistochemical staining for JunD in acini, metaplasia and cancer examples for KC (n=3), KC;iASPP^Δ8/Δ8^ (n=3) and KPC (n=3) pancreata. Scale bar, 50 µm.

(I) Representative immunohistochemical staining for phospho-p65 (Ser276) in acini, metaplasia and cancer examples for KC (n=3), KC;iASPP^Δ8/Δ8^ (n=3) and KPC (n=3) pancreata. Scale bar, 50 µm.
